# Overexpression of the brassinosteroid biosynthetic gene *DWF4* in *Brassica napus* simultaneously increases seed yield and stress tolerance

**DOI:** 10.1038/srep28298

**Published:** 2016-06-21

**Authors:** Sangita Sahni, Bishun D. Prasad, Qing Liu, Vojislava Grbic, Andrew Sharpe, Surinder P. Singh, Priti Krishna

**Affiliations:** 1 Department of Biology, The University of Western Ontario, London, ON, N6A 5B7, Canada; 2 Commonwealth Scientific and Industrial Research Organization, Agriculture, PO Box 1600, Canberra, ACT 2601, Australia; 3 National Research Council Canada, Plant Biotechnology Institute, 110 Gymnasium Place, Saskatoon, SK S7N 0W9, Canada; 4 School of Environmental and Rural Sciences, University of New England, Armidale, NSW 2350, Australia

## Abstract

As a resource allocation strategy, plant growth and defense responses are generally mutually antagonistic. Brassinosteroid (BR) regulates many aspects of plant development and stress responses, however, genetic evidence of its integrated effects on plant growth and stress tolerance is lacking. We overexpressed the *Arabidopsis* BR biosynthetic gene *AtDWF4* in the oilseed plant *Brassica napus* and scored growth and stress response phenotypes. The transgenic *B. napus* plants, in comparison to wild type, displayed increased seed yield leading to increased overall oil content per plant, higher root biomass and root length, significantly better tolerance to dehydration and heat stress, and enhanced resistance to necrotrophic fungal pathogens *Leptosphaeria maculans* and *Sclerotinia sclerotiorum.* Transcriptome analysis supported the integrated effects of BR on growth and stress responses; in addition to BR responses associated with growth, a predominant plant defense signature, likely mediated by BES1/BZR1, was evident in the transgenic plants. These results establish that BR can interactively and simultaneously enhance abiotic and biotic stress tolerance and plant productivity. The ability to confer pleiotropic beneficial effects that are associated with different agronomic traits suggests that BR–related genes may be important targets for simultaneously increasing plant productivity and performance under stress conditions.

Brassinosteroids (BRs) are plant steroid hormones, which play essential roles in plant growth and development^1^,^2^, and also confer tolerance to a wide range of abiotic stresses^3^,^4^. The BR signalling pathway is one of the best-understood signal transduction pathways in the plant hormone field. BR is perceived by the membrane-localized BRASSINOSTEROID INSENSITIVE1 (BRI1), which upon ligand binding heterodimerizes with BRI1-ASSOCIATED RECEPTOR KINASE (BAK1), resulting in sequential reciprocal receptor transphosphorylation to enhance signal output. BR activated signalling by BRI1 inactivates the negative regulator BRASSINOSTEROID INSENSITIVE2 (BIN2), which in turn leads to activation of two closely related transcription factors BRI1-EMS SUPPRESSOR1 (BES1) and BRASSINAZOLE-RESISTANT1 (BZR1) and expression of an array of BR-regulated genes^5^,^6^. BAK1 and its homologs are also a required component of pathogen/microbe-associated molecular patterns (PAMPs/MAMPs)-triggered immunity and Mi-1-mediated resistance to insect herbivores^1^,^2^.

Important roles for BRs in plant developmental processes include regulation of photomorphogenesis, seed germination, flowering, senescence, pollen tube elongation, xylem differentiation and root growth^1^,^2^. Studies on BR effects have focused on either a single plant trait or a subset of traits. For example, ectopic as well as organ-specific overexpression (OE) of *DWF4* encoding a C-22 hydroxylase that catalyzes a rate-determining step in BR biosynthesis, was noted to increase inflorescence height, branch numbers and the number of seeds per plant^7^,^8^, as well as seed weight^9^ in *Arabidopsis*, tobacco and rice. While these observations established that the BR biosynthetic pathway and the role of *DWF4* are conserved across plant species of diverse lieages^10^, it remains unknown how BR-mediated shoot growth outputs interface with root phenotypes, and with plant performance under different stress regimes.

The role of BR in conferring tolerance to a wide range of abiotic stresses, such as high and low temperatures, salinity and drought have been described in numerous studies^3^,^4^. Most of the studies focused on BR effects on plant stress responses have been conducted using exogenous BR. In one study a single mutant allele of *GSK1* (*GSK3/SHAGGY-LIKE PROTEIN KINASE1*), an orthologue of BIN2, was noted to be more tolerant to abiotic stresses than non-transgenic segregants, leaving a small amount of doubt that some of the phenotypes may arise from a mutation in a locus other than *GSK1*^11^. Thus, additional genetic evidence is required in support of BR’s role in stress tolerance.

Exogenously administered BR also increased general plant resistance against pathogens^4^,^12^,^13^, but in some studies BR appeared to inhibit the well-studied MAMP flg22-induced responses^14^,^15^, even rendering plants hypersusceptible to root pathogens^16^. These conflicting findings indicate that BR’s role in plant growth and immunity is a complex phenomenon. More importantly, genetic evidence for integrated BR effects on plant growth and immunity are lacking.

Thus, while the role of BR in plant growth and development is well established, it is is currently not known which growth traits can be simultaneously influenced by BR and how these growth traits interface with abiotic and biotic stress tolerance. We chose to address these questions through ectopic overexpression of *AtDWF4* in the economically important oilseed crop plant *Brassica napus*. The transgenic *B. napus* plants were characterized with increased seed yield, higher root biomass and root length, significantly better tolerance to dehydration and heat stress, and increased resistance to the two necrotrophic fungal pathogens tested, as compared to WT. Microarray analysis of transcriptomic changes in the transgenic plants identified, in addition to BR-mediated growth responses, signatures of both biotic and abiotic stress responses. Overall, a net positive impact on plant productivity and performance was achieved by manipulation of a single BR biosynthetic gene, emphasizing modulation of these traits by BR at a higher level in the hierarchical response system of plants. These findings also provide a strong base for developing BR-based breeding programs for crop improvement.

## Results

### *AtDWF4* OE increases branching, silique formation and seed yield

**Figure 1:**
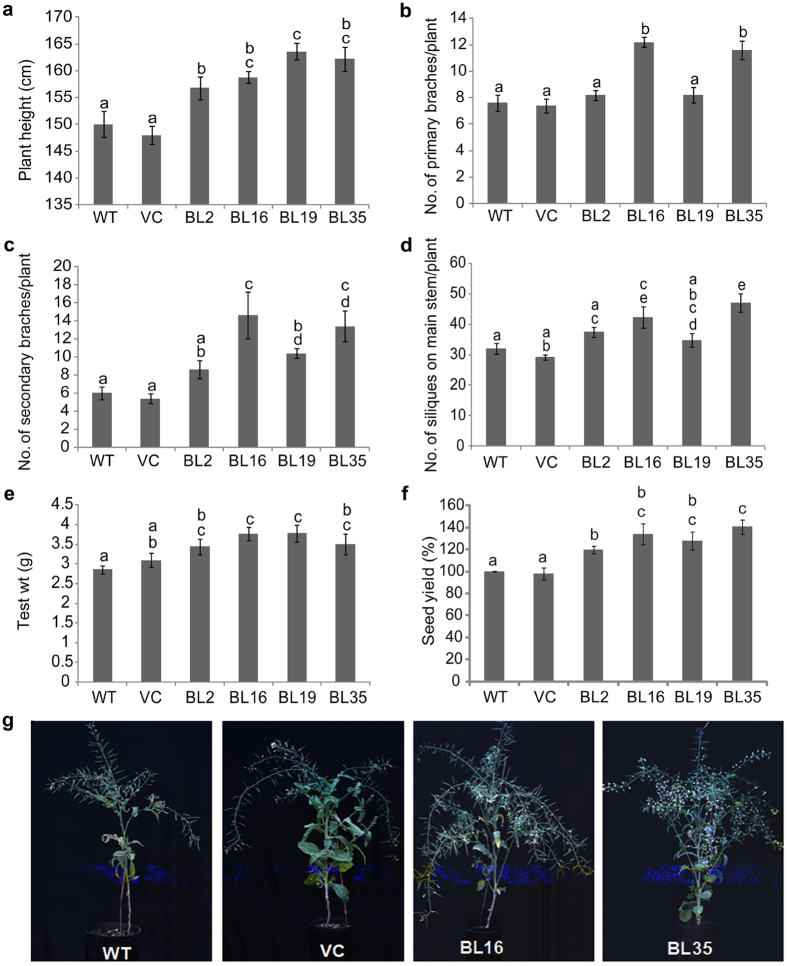
Growth and yield-attributing traits. (**a**) Plant height. (**b**,**c**) Number of primary and secondary branches/plant, respectively. (**d**) Number of siliques on main stem. (**e**) Test weight (weight of 1000 seeds). (**f**) Seed yield (%). (**g**) Phenotypes at 90 DAT.

The *AtDWF4* full-length coding sequence was placed under the control of CaMV 35S promoter and introduced into the *B. napus* genome. Four homozygous transgenic lines BL2, BL16, BL19 and BL35 with a single transgene insert and varying levels of transgene expression (Supplemental Fig. S1) were chosen for phenotypic evaluations. WT, vector control (VC) and transgenic seedlings were first grown in Magenta™ vessels for 14 days, and subsequently transplanted in soil-filled pots and grown to maturity in the greenhouse. Transgenic plants at 90 days after transplanting (DAT) were 4.5–9% taller than WT (Fig. 1a), had larger leaves, slightly longer petioles, and more branches as compared to WT plants (Fig. 1b,c). BL16 and BL35 lines had more branches per plant, and greater number of siliques on the main stem (Fig. 1d). A comparison of the average weights of 1000 similar sized seeds showed ~32 and 22% increase, respectively, in BL16 and BL35 as compared to WT (Fig. 1e). Due to these phenotypic changes, seed production per plant was increased between 20–40% in different transgenic lines (Fig. 1f). The increase in overall vegetative biomass (Fig. 1g) and seed production is consistent with results in *Arabidopsis*, tobacco^7^,^8^ and rice^9^. These results confirm conservation of *AtDWF4* function in *B. napus*, as well as conservation of endogenous BR pathways and responses in *B. napus.* The high level of relatedness (90% amino acid identity) between *AtDWF4* and the most closely related *BnDWF4* orthologue also corroborates conservation of the DWF4 enzyme in both plant species.

### *AtDWF4* OE does not alter seed oil content and composition

Seed oil content is an important agronomic trait for *B. napus* as it is a source of edible (canola) and industrial oils, including biofuel. There were no significant differences in the lipid content (Supplemental Fig. S2) and composition [palmitic (C16:0), stearic (C18:0), oleic (C18:1), linoleic (C18:2) and linolenic (C18:3) acids] of transgenic and control plant seeds (Supplemental Table S1). As further confirmation, we also analyzed the seeds of oleosin promoter driven *AtDWF4* expressing *Arabidopsis* plants^17^, but found no significant change (Supplemental Fig. S3 and Supplemental Table S2). These data suggest that while efficient photosynthesis may lead to increases in biomass growth^9^, the mechanisms that regulate conversion of sucrose, the main carbon source for storage oil synthesis, to triacylglycerol in oil-storing seeds are tightly controlled. Although the oil content per gm seed weight remained the same, the total oil yield per plant was increased due to enhanced seed yield.

### *AtDWF4* OE leads to a larger shoot and root system

**Figure 2:**
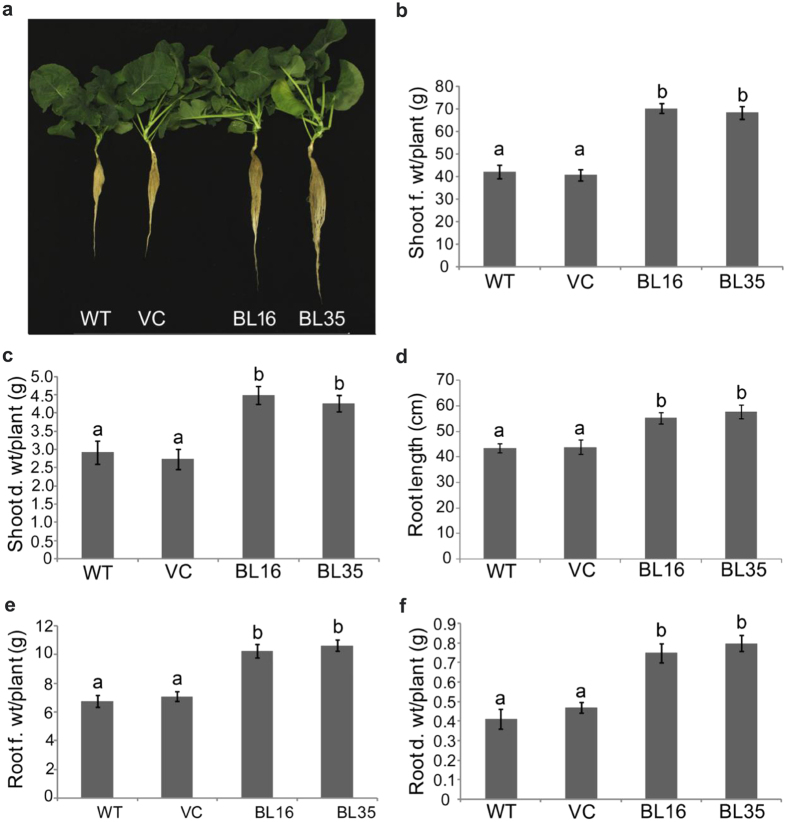
Phenotypes of hydroponically grown plants. (**a**) Seedlings at 30 DAT to glass jars. (**b**) and (**c**) Shoot fresh (**f**) and dry (**d**) weight (wt), respectively. (**d**) Root length. (**e**,**f**) Root f. and d. wt, respectively.

To accurately assess shoot and root growth, WT, VC, and transgenic lines BL16 and BL35 most representative of BR phenotypes, were grown in a hydroponic system for 30 days. The transgenic plants maintained a larger shoot system with greater leaf size and petiole length compared to control plants (Supplemental Fig. S4). For example, leaf length and width, and petiole length in BL16 plants was increased by ~34, 31 and 25%, respectively, which was accompanied by ~67 and 54% increase in the fresh and dry shoot weights, respectively, over WT (Fig. 2a–c). Interestingly, BL16 and BL35 plants also showed ~27 and 33% increase in root length (Fig. 2a,d), 52 and 58% increase in fresh root weight (Fig. 2e), and 83 and 95% increase in dry root weight (Fig. 2f), respectively. A larger root system was also observed in soil grown transgenic plants (Supplemental Fig. S5). The *AtDWF4*-mediated simultaneous allocation of biomass to shoot and root suggests advantage to the plants in acquiring the most critical resources, light and water, and has implications for agricultural production.

### *AtDWF4* OE enhances drought tolerance

**Figure 3:**
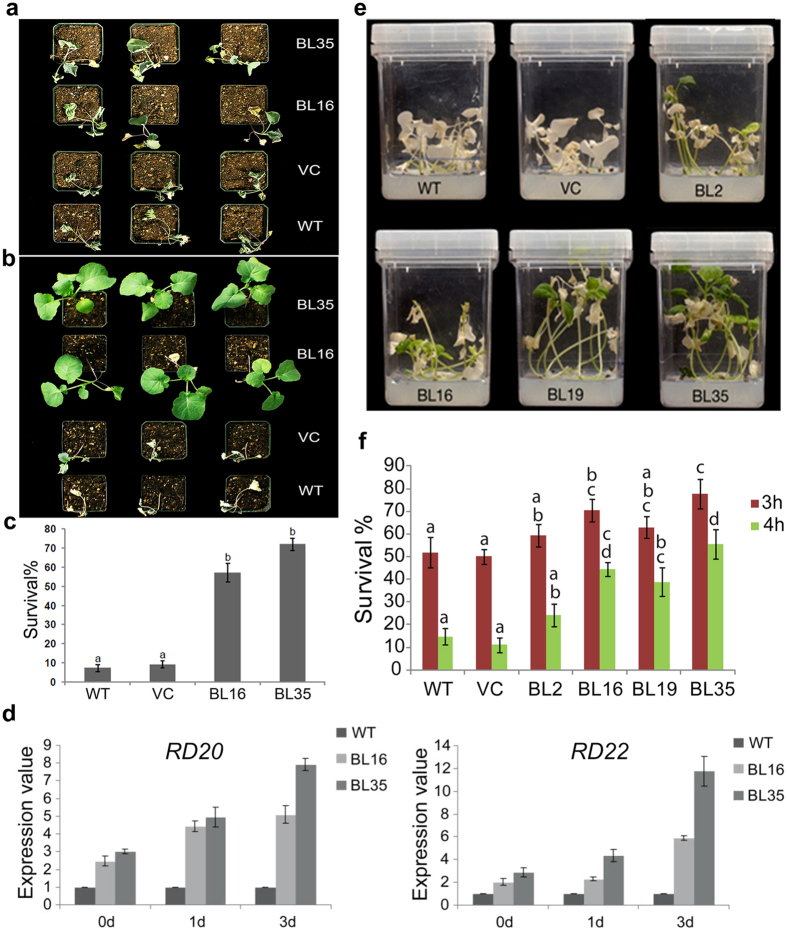
Seedling survival after water and heat stress. (**a**) Seedlings drought-stressed for 12 days. (**b**) Drought-stressed seedlings re-watered for 7 days. (**c**) Percent survival after re-watering. (**d**) qRT-PCR analysis of *RD20* and *RD22* in drought-stressed seedlings. (**e**) Seedlings exposed to 45 °C for 4 h and then recovered at 20 °C for 7 days. (**f**) Percent survival.

Earlier we demonstrated that exogenous BR renders *B. napus* and *Arabidopsis* seedlings to be more drought tolerant than untreated seedlings^18^. To assess the response of *AtDWF4* seedlings, 4-week-old plants grown in plastic pots containing Pro-Mix BX were deprived of water for 12 days and their phenotypes were scored after resupplying water for 7 days. Figure 3a,b represent the phenotypes of water-deprived plants and of plants that subsequently received water, respectively. The transgenic plants had up to 10-fold higher survival rate (57 to 72%) as compared to WT plants (7%) (Fig. 3c). The survival advantage of transgenic seedlings was also reflected in the markedly different root and shoot weights following dehydration stress and subsequent re-watering (Supplemental Figs S6 and S7). For example, the average fresh and dry root weights of BL35 seedlings were 1.6 and 1.8-fold higher, respectively, than those of WT before stress, but this difference was escalated to ~16-fold and 12-fold, following stress. Although growth was stalled in all surviving plants, the transgenic plants continued to retain bigger sizes over the surviving control plants (Supplemental Fig. S7). The root/shoot ratio of the dry weight of transgenic plants was lower than the control plants before dehydration stress, but after stress and re-watering, the ratio in transgenic plants was higher. In case of BL35 the ratio was more than 2 times of WT and VC plants receiving the same treatment, indicating preferential allocation of resources to roots during stress/recovery in the transgenic plants.

To see if the *AtDWF4* effect on drought tolerance involved the canonical drought responses, the expression of dehydration stress-responsive marker genes *RD20* (*RESPONSIVE TO DEHYDRATION20*) and *RD22*^19^ was analyzed using qRT-PCR and correlated with drought tolerance of the genotypes. *RD20* and *RD22* were expressed at 2.5 to 3-fold higher levels in BL16 and BL35 over WT in the absence of stress, but this difference escalated to 5 to 8-fold for *RD20,* and 6 to 12-fold for *RD22*, respectively, by 3 days of drought stress (Fig. 3d), indicating a highly dynamic stress response in transgenic seedlings *vs* WT. The recent observation that barley semi-dwarf allelic mutants with decreased BR synthesis due to mutation in *HvDWARF* are compromised in growth, proline, sucrose and ABA accumulation in response to drought further supports a role for BR in drought stress tolerance^20^.

### *AtDWF4* OE enhances basal thermotolerance

Heat stress slows vegetative growth and is a major cause of reduced reproductive yield in *B. napus*^21^. Exogenously supplied BR increased basic thermotolerance of *B. napus* seedlings^22^,^23^. Two-week-old WT, VC, and transgenic seedlings grown in Magenta™ vessels were exposed to 45 °C for 3 and 4 h, and then allowed to recover at 20 °C for 7 days. Transgenic seedlings survived to different levels following heat stress (HS) treatment for 4 h, but in each case survival was better than WT and VC (Fig. 3e,f). Approximately 77 and 55% of BL35 seedlings survived exposure to 45 °C for 3 and 4 h, respectively, as compared to 52 and 15% of WT seedlings. Consistent with the exogenous BR effects, Heat Shock Protein (Hsp)101 and Hsp90, markers of thermotolerance, accumulated to much higher levels in transgenic seedlings than in WT seedlings^22^,^23^.

### *AtDWF4* OE suppresses fungal infection symptoms

**Figure 4:**
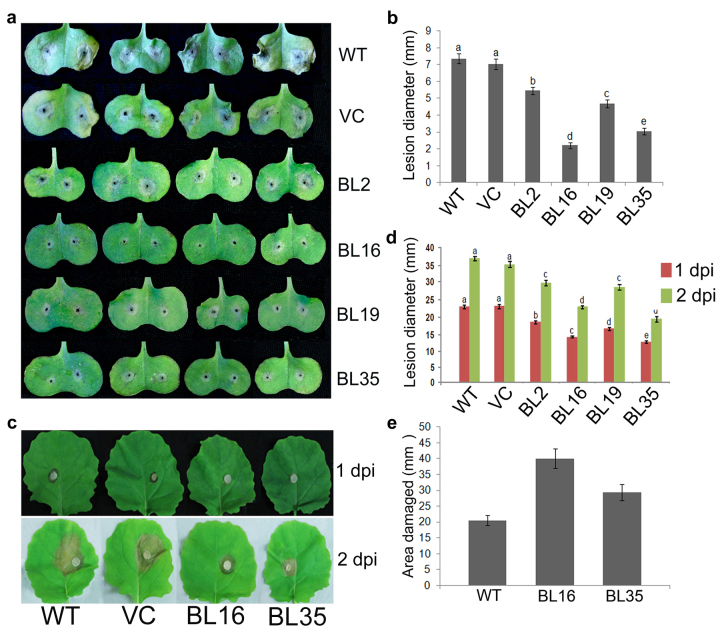
Test for pathogen and pest resistance. (**a**) Necrotic lesions and (**b**) lesion diameter on cotyledons challenged with *L. maculans* (7 dpi). (**c**,**d**) Necrotic lesions and lesion diameter at 1 and 2 dpi, respectively, on leaves challenged with *S. sclerotiorum*. (**e**) Leaf area damaged by spider mite feeding (4 dpi).

The fungal pathogen *Leptosphaeria maculans* causes blackleg or stem canker on Brassica crops and constitutes a major threat to the cultivation of Brassicas in many parts of the world. Described as a hemibiotroph, *L. maculans* undergoes alternating periods of biotrophic and necrotrophic growth during the infection life cycle^24^. Ten cotyledons from each genotype were inoculated on two sites and the necrotic lesion diameters were measured 7 days post infection (dpi). The mean lesion diameter in cotyledons of the four transgenic lines ranged from 2.2 to 5.4 mm and was considerably smaller than of WT plants (7.3 mm) (Fig. 4a,b). Transgenic lines BL16 and BL35 showed ~70 and 59% reduction in lesion diameter, respectively, compared to WT, and were more resistant than the other two lines in this order. In WT and VC, the symptoms also included yellowing and collapse of the tissue next to the gray lesions, which were not detected in transgenic plants.

*Sclerotinia sclerotiorum* is another necrotrophic, non-host-specific fungal pathogen that can cause disease in more than 400 plant species, including members of the *Brassicaceae* family^25^. In the detached leaf assay, the 3^rd^ and 4^th^ leaves of plants were removed and inoculated with *S. sclerotiorum* mycelial plugs. The transgenic lines exhibited delayed lesion occurrence and significantly smaller lesion sizes compared to WT and VC at 2 and 3 dpi (Fig. 4c,d). Necrosis was visible in WT and VC as early as 1 dpi, but in transgenic plants it became apparent at either 2 or 3 dpi. Lesion sizes in BL16 and BL35 leaves at 3 dpi were 38 and 47% smaller, respectively, than in WT. A similar reduction in lesion diameter (42 and 51%, respectively) in BL16 and BL35 leaves was also observed in the whole plant leaf inoculation assay (Supplemental Fig. S8). These results indicate that *AtDWF4-*expressing transgenic lines are significantly more resistant to the fungal pathogens tested than WT and VC.

A large number of insect pests attack Brassica species, including beetles, caterpillars, aphids, thrips and spider mites. Brassicas, like other plants, respond to the cues generated by herbivores by both constitutive and inducible defense mechanisms^26^. The BR-herbivore relationship was explored by infesting the 2^nd^ and 3^rd^ leaves of 14-day-old *B. napus* seedlings with 500 active spider mites/leaf, and the damage (chlorotic spots that appear in association with feeding) was compared with damage inflicted on WT seedlings at 4 dpi. The damaged leaf area in the two transgenic lines, ranging from 30 to 40 mm^2^, was significantly larger than the average 20 mm^2^ in the WT plants (Fig. 4e). Thus, the two transgenic lines tested were more vulnerable to feeding by mites.

### Effects of *AtDWF4* OE on SA and JA levels

Typically SA and JA are considered as primary hormones associated with defense against pathogens and pests, respectively, though the final defense response output is more a result of hormonal interactions^27^. Effects of BR, if any, on SA and JA^28^ levels are not well established. We found that exogenous BR increases JA levels in *Arabidopsis*^29^. In the present study, SA and JA levels were analyzed in leaves of 2-week-old WT, VC, transgenic lines, and 24-epibrassinoslide (EBR)-treated WT *B. napus* seedlings (Supplemental Fig. S9). With the exception of transgenic line BL16, all other genotypes had SA levels similar to control plants. JA in *B. napus* leaves was present at low levels, ranging from 1.3 to 9 ng/g fresh weight. BL2, BL19 and EBR-treated WT seedlings had ~1.8, 3 and 2-fold higher JA levels, respectively, while BL35 and BL16 had ~1.5 and 2-fold lower JA levels, respectively, as compared to WT.

### *AtDWF4* OE leads to enhanced defense gene expression

We used the *B. napus* 50-mer oligo array composed of 15,000 unique gene-specific probes^30^ to detect gene expression differences in BL16 and BL35 *vs* WT, and EBR-treated WT *vs* untreated. Considering that BR produces only modest changes in gene expression^31^, differentially expressed genes with 1.5-fold change cutoff (p-values ≤ 0.05) were identified. A total of 2,639 genes significantly changed their expression in all genotypes compared to untreated WT. Roughly equal numbers comprising of 810, 875 and 954 genes were differentially expressed in BL16, BL35 and EBR-treated seedlings, respectively. Of these, 186 differentially expressed genes (37 up and 127 downregulated) were common between BL16, BL35 and EBR-treated, and an additional 72, 132 and 146 were common between BL16 and EBR-treated, BL35 and EBR-treated seedlings, and BL16 and BL35, respectively (Supplemental Fig. S10).

The reliability of microarray data was evaluated by qRT-PCR of differentially expressed genes in BL16 and BL35 compared to WT (Supplemental Fig. 11a), and in EBR-treated WT plants compared to untreated WT (Supplemental Fig. 11b). A high degree of correlation (microarray BL16 *vs* QBL16, *r* = 0.93; microarray BL35 *vs* QBL35, *r* = 0.96; EBR *vs* QEBR, *r* = 0.87) was observed, indicating high level of consistency of data by the two methods.

### Functional classification of differentially expressed genes

Differentially expressed genes were functionally classified using MapMan software^32^. Bins carrying more than five differentially expressed genes for each sample are shown in Supplemental Fig. S12. Most notable is the positive effect of exogenous EBR on cell wall-related genes. The RNA, protein synthesis, stress, and hormone metabolism bins were overrepresented in all three samples (BL16, BL35, EBR-treated WT).

Well-characterized BR-responsive genes, such as expansins (EXP), xyloglucan endotransglucosylases (XET)/hydrolases (XTH), pectinesterases, glutathione S-transferases, glycine-rich proteins and ethylene biosynthesis-related^31^,^33^ were differentially expressed across samples (Table 1, Supplemental Fig. S12). Within the ‘Cell wall’ Bin, the most prominent in expression include upregulation of *XTH17, 18, 19* and *33*, *EXPR3*, and pectinestersae family proteins *At2g45220* and *At4g02330,* and consistent downregulation of *PGIP1* (POLYGALACTURONASE INHIBITING PROTEIN 1) and *PGIP2*. These various cell wall modifying enzymes modulate cell wall extensibility and plasticity during both developmental and stress response programs^34^. Consistent with the overlap in gene targets between auxin and BR^30^, four auxin-responsive genes (*At4g38840, At5g18060, At4g34810, At5g35735*) were common to all three samples (Table 1). Together, these data strongly endorse that the phenotypes and associated molecular changes in transgenic lines are due to enhanced BR levels.

A striking feature within the gene expression dataset was the upregulation of numerous defense-related genes. Confirmation by qRT-PCR of *PR1*, *PR5*, *TIR1, PDF1.2b,* and *PDF1.3* showed ~14, 13, 15, 1.5 and 7-fold induction in BL16, and 26, 26, 28, 1.5 and 20-fold induction in BL35, respectively (Supplemental Fig. 11a). *PR5*, *PDF1.2b* and *PDF1.3* also showed ~3, 2 and 1.9-fold induction, respectively, in EBR-treated seedlings (Supplemental Fig. 11b). Additional examples of biotic stress response were genes encoding disease resistance proteins/related, chitinases, cysteine proteinases, β-1,3-glucanases, trypsin and protease inhibitor family proteins, defense-related metabolic enzymes (aldose reductase, glucosyl hydrolase family, dehydroascorbate reductase), antioxidant proteins/related (glutathione S-transferase, superoxide dismutase, glutaredoxin family, peroxidase) and WRKY transcription factors (Table 2). In some cases different members of the same gene family were differentially expressed in the three samples, as exemplified by the *PDF* genes (Table 2).

Amongst the differentially regulated abiotic genes, heat stress-responsive genes were predominant, including several *DnaJ* gene family members, *HSP17.6* and *HSP17.4*, and single *Hsp90, Hsp70,* and *Hsp60* gene family members. Some drought, cold and salt stress -responsive genes were also present in the ‘Stress’ MapMan bin.

Within the common downregulated genes (Table 1), the consistent downregulation of JA biosynthesis (*LOX2,3*&*4*, *OPR1,2*&*3*) and JA signaling (*JAZ*) genes in BL16, BL35 and EBR-treated seedlings is noteworthy. Although JAZ proteins act as repressors of JA response, they are also upregulated by JA possibly to provide a negative feedback loop^35^. Reduced expression of these genes under reduced JA synthesis could then be anticipated.

Consistent with our previous observation of BR effects on translation apparatus components^23^, genes encoding eukaryotic elongation factors eEF1 beta and eEF-1B gamma were upregulated 1.6 to 3.8-fold in all three samples (Table 1). Additionally, initiation factor eIF3 gamma subunit family, eIF-3E, and eEF-1-alpha putative were upregulated in some samples (Supplemental Fig. S12).

### Genes maximally upregulated by BR in AtGenExpress dataset

**Figure 5:**
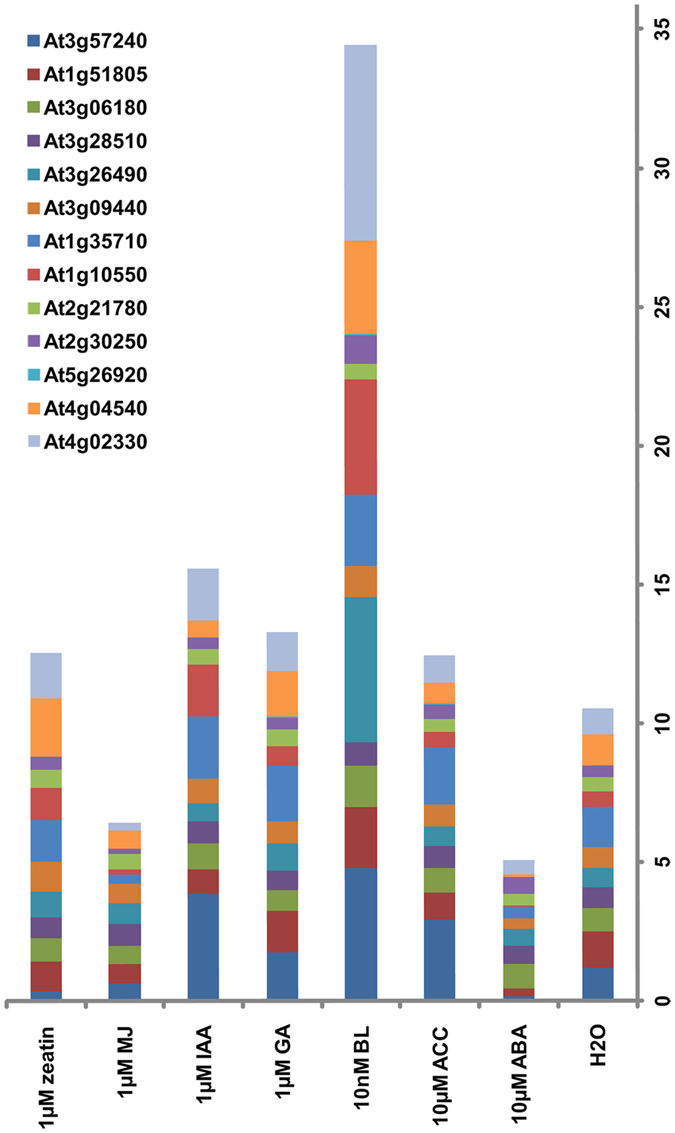
Genes maximally induced by BR in AtGenExpress. Absolute expression values in response to 3 h hormone treatment were retrieved by AVT for genes upregulated (≥2-fold) in BL16 and BL35 by microarray analysis. The height of the bar of any one color indicates fold-change in response to a hormone for that specific gene. The numbers on the Y-axis serve as a scale for fold-change.

Since the BR response output involves interactions with other hormones^36^, we wished to point directly to BR response genes. Genes showing ≥2 fold upregulation in one or both transgenic lines were analyzed for hormone responses in the public database AtGenExpress. Thirteen genes had maximal induction by BR as compared to other hormones (Fig. 5, Supplemental Table S3). According to TAIR, five of these genes are associated with defense, three with abiotic stress, and the others with light stimulus, cell wall modification, kinase activity and protein synthesis. The detection of several defense-related genes, including *WRKY* genes, within the BR response genes lists (Table 1 and Supplemental Table S3), suggests that BR’s role in modulating biotic stress responses is authentic.

WRKY transcription factors are regulatory proteins that function in both biotic and abiotic stress responses^37^. The identification of a total of eight differentially expressed *WRKY* genes (Table 2), of *WRKY25* as a putative direct BR response gene (Supplemental Table S3), of *WRKY18* and *WRKY11* in the gene list of upregulated and downregulated in all three samples, respectively (Table 1) suggests that WRKY proteins are important components of BR-mediated defense against pathogens and salinity tolerance.

### Differentially expressed genes as putative targets of BZR1/BES1

We considered genes maximally upregulated by BR (Supplemental Table S3) and those upregulated and downregulated in all 3 samples (Table 1) as authentic BR response genes. A search of the gene promoters against BZR1 (CGTGT/CG)^5^ and BES1 (CANNTG, CACGTG and CACTTG)^6^ binding sites identified majority of these genes as putative targets of BZR1/BES1. Though experimental validation is needed, these data indicate a high probability that regulation occurs via BZR1/BES1 signaling. Due to the presence of many stress response genes within this dataset, it is tentatively concluded that BR mediates stress responses, at least in part, via BZR/BES1. The recent observation that several *BZR*-related genes in *Brassica rapa* are induced by drought, cold and salt supports their role in stress-related gene expression^38^.

## Discussion

This study demonstrates that OE of a single BR biosynthetic gene *AtDWF4* in the economically important oilseed *B. napus* could simultaneously increase crop yield and stress tolerance. This finding is of particular importance as most stress hormones such as ABA^39^, JA^40^ and SA^41^ inhibit growth at elevated levels as a strategy for partitioning metabolic resources between the competing processes of growth and stress response.

The question arises–what mechanisms may BR employ to confer these pleiotropic beneficial effects? Likely BR’s ability for this lies in its widespread, crucial, and higher-level coordinative roles in cellular processes. We discuss three such possibilities: 1) BR’s ability to modulate basic structure and functions of the cell; 2) BR’s ability to directly and indirectly boost stress responses; and 3) BR’s ability to mobilize resources for growth regulation under stress conditions. The first point derives from BR’s positive roles in protein synthesis^23^, cell wall biogenesis and modifications^42^, xylem differentiation^43^, photosynthesis/chloroplast functions^44^,^45^ and the flow of assimilate from source to sink^9^. These themes recur in the present study in that several genes encoding translation eEFs and eIFs, cell wall modifying EXPs, XETs/XTHs, and pectinesterases, photosynthesis related chlorophyll A-B binding family proteins, and cytochrome P450 71A16 and other proteins, were upregulated in *AtDWF4* transgenic as well as EBR-treated seedlings. Similarly, ‘RNA’ and ‘protein’ MapMan bins were most prominent in the three samples (Supplemental Fig. S12), indicating that BR affects these processes. It is noteworthy that BR’s impact on protein synthesis^23^ was also noted in rats fed with BR where body weight and muscle mass gain were linked with BR’s ability to stimulate protein synthesis and inhibit protein degradation^46^. Thus, with positive impacts on many basic plant cellular processes and structural changes, BR promotes vigor, which would lend prior advantage in adaptation and defense against environmental stresses.

In regards to the second point, the present study serves as genetic proof in support of BR’s ability to trigger a more vigorous stress response program as compared to controls^18^,^22^,^29^,^36^. Even with a limited set of genes represented on the Brassica microarray, a distinct defense response signature was noted in *AtDWF4* transgenic lines under no-stress conditions (Table 2). Some abiotic stress response genes were downregulated under no-stress conditions, but in response to drought and heat stress the transgenic lines mounted a more vigorous stress response than WT (Fig. 3). Thus, it seems that BR incorporates a dynamic and flexible program for balancing growth and stress responses, with some genes being ‘ON’ prior to stress and other genes primed to be switched ‘ON’ when the need occurs. Following the same line of reasoning, the downregulation of several JA biosynthesis and signalling genes (*JAZ*) in all three samples (Table 1) and the slightly lower, though statistically insignificant, JA levels in BL16 and BL35 (Supplemental Fig. S9) may be mechanisms of avoiding or balancing the inhibitory effect of JA on growth. These observations coupled with the identification of several JA synthesis (*LOX2*, *LOX3*, *AOS* and *OPR3*) and JA signaling genes (*JAZ* genes and the positive regulator) as putative targets of BZR1/BES1 (Table 1), suggest BR to function as a key regulator of JA homeostasis and JA signaling to maintain a balance between growth and defense responses.

The third point, ie., BR’s ability to mobilize resources during stress is supported in the observation that BL16 and BL35 showed an increase in dry weight root/shoot ratio over WT in response to dehydration stress, while under no-stress condition the WT showed a higher ratio (Supplemental Fig. S6). This kind of adaptive plasticity must involve metabolic programming. While it is known that BR enhances plants’ carbon assimilatory capacity, which in turn increases sink strength, more focused studies are needed to understand how BR controls source-sink relationships during stress episodes.

The *AtDWF4* transgenic lines showed greater resistance to the two fungal pathogens tested, but slightly higher preference for spider mite feeding than did WT (Fig. 4). Defense against *L. maculans* involves both race-specific resistance based on the gene-for-gene interaction concept^47^ and quantitative resistance involving a basal defense against pathogen attack^48^. Responses of primary importance appear to culminate in physical barriers that prevent the spread of the pathoge^49^. Defense against *S. sclerotiorum* also involves formation of a defensive barrier, which in part derives from lignin deposition to increase cell wall rigidity^50^, as well as the hypersensitive reaction (HR)^51^.

The mechanisms by which BR promotes resistance to fungal pathogens may consist of 1) a higher basal defense preparedness, as indicated by the defense response signature identified in this study; 2) ability to remodel cell wall^52^ to provide a defensive barrier to the pathogen, and 3) ability to modulate cellular concentrations of reactive oxygen species (ROS)^53^,^54^. The differential regulation of numerous ROS-associated genes encoding glutathione S-transferase, superoxide dismutase, glutaredoxin family, thioredoxin, peroxidase, dehydroascorbate reductase reinforces the BR and ROS link. That BR activates ROS generation to induce defense gene expression, cell wall strengthening and programmed cell death to restrict pathogen spread, is an attractive idea that merits further study. The identification of a number of cell wall related genes in microarray analysis (Supplemental Fig. S12) correlates with BR’s function in cell wall modification^52^, a process that is increasingly being correlated with plant stress tolerance^34^,^55^. A detailed analysis of the cell wall composition of the transgenics in the future will allow us to make more definitive conclusions.

**Figure 6:**
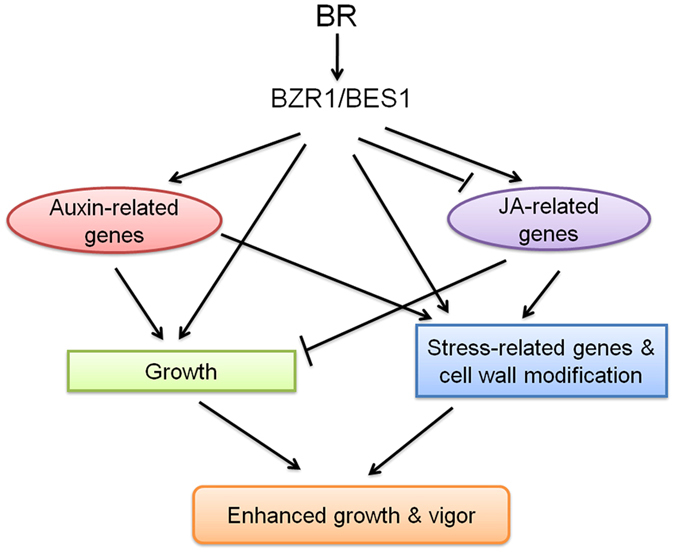
Model depicting BR-mediated changes via BES1/BZR1 signaling, leading to enhanced growth and vigor. Auxin and JA signatures were prominent in the present study. BR interacts with auxin to promote growth, and downregulates JA-related genes in the absence of stress to balance growth and stress responses. BR impacts cell wall composition and stress responses integrated with growth for built-in resilience, and primes plants for enhanced defence when challenged.

There is a growing realization that abiotic and biotic stresses trigger overlapping response pathways in plants^56^. That BR modulates such overlapping responses can be seen in the present study in differentially expressed *WRKY*, *PR*, *HSP*, protein synthesis, calcium signaling and ROS-associated genes, as well as several other genes responsive to both types of stresses. It is suggested that BR’s program for stress tolerance via these and other genes will be different from the specific and definite responses that have been defined in literature for specific plant-signal interactions since BR mechanisms will include cross stress regulation and integration with growth. Based on the results of this study, a model incorporating growth and stress responses mediated directly by BR and via interaction with auxin and JA, leading to enhanced plant growth and vigor is depicted in Fig. 6.

In summary, BR’s higher-level role in regulating many different physiological processes appears to culminate in producing multiple beneficial effects in the *AtDWF4 B. napus* plant. From an agricultural perspective, BR-related genes could serve as breeding targets for crop improvement.

## Methods

### Generation of *AtDWF4* transgenic plants

The *A. thaliana DWF4* cDNA (Arabidopsis Biological Resource Center, clone no. U13551) was PCR amplified and cloned into pRTL-GUS. The expression cassette (CaMV 35S promoter, *AtDWF4* coding region, and nos terminator) was transferred to pCAMBIA 2301 and the resulting construct was introduced into *B. napus* using the cotyledon/petiole co-cultivation method^57^. A total of 28 kanamycin-resistant T1 transgenic plants were obtained. DNA from T2 generation plants of 12 independent transgenic lines was analyzed by PCR and Southern blotting (data not shown) for detecting the presence and number of integration sites of the transgene, respectively. Homozygous lines BL2, BL16, BL19, and BL35 with single inserts and varying levels of transgene expression (Supplemental Fig. S1) were chosen for the study. Transgenic VC lines were generated using the empty pCAMBIA 2301 vector.

### qRT-PCR

Total RNA was isolated from leaf tissue using the SV Total RNA Isolation System (Promega). First strand cDNA was synthesized using QuantiTect Reverse Transcription Kit (Qiagen). qRT-PCR details are provided in Supplemental methods (Method S1).

### Plant growth and phenotypic analysis

Seeds of WT, VC and transgenic lines were grown in pots filled with Pro-Mix BX (Premier Horticulture) under greenhouse conditions supplemented with GE Lucalox^®^400W (General Electric) to give a 16 h photoperiod. Plant growth traits (height and number branches) were measured at 90 DAT. Siliques were counted only on the main stem. Seeds were harvested from whole plants, air-dried for a week and weighed. The weight of 1000 seeds was determined as test weight.

For hydroponic growth, surface sterilized *B. napus* seeds were germinated in petri dishes for 48 h. Seedlings were transferred into pots filled with industrial sand and grown at 20 °C under 16/8 h light/dark cycle with light intensity of 125 μmol m^–2^s^–1^. Pots were watered once daily with 1X Hoagland’s nutrient solution^58^. After 10 days seedlings were transferred into 1.4 L glass jars (one seedling per jar) filled with Hoagland’s nutrient solution and connected to an aeration system (Maxima 805 Air Pump, Rolf C. Hagen Inc.). The nutrient solution was changed every 3^rd^ day. Data were recorded after 30 days. For dry weight, plant tissue was dried at 60 °C until constant weight was reached. The experiment (n = 4 plants/genotype) was set up in a randomized complete block design (RCBD) and repeated twice.

### Stress assays

Four-week-old seedlings, grown in plastic pots (8 × 7.5 cm^2^) containing the same amount of Pro-Mix BX per pot, were subjected to drought stress by withholding water for 12 days and then allowed to recover by watering them regularly for next 7 days. Data was recorded following the recovery phase. Control plants were watered for the entire duration. Experiments (n = 18 plants/replicate of WT, VC, BL16 and BL35) were laid out under RCBD and repeated three times. Heat stress was given as described by Dhaubhadel *et al.*^22^. Two-week-old *B. napus* seedlings grown on MS medium in Magenta vessels at 20 °C under a 16/8 h light/dark cycle and a light intensity of 80 μmol m^–2 ^s^–1^, were exposed to 45 °C for 3 and 4 h, and then returned to the growth chamber set to 20 °C to recover for a period of 7 days. After recovery, seedlings that continued to grow were counted. Expression profiles of Hsp90 and Hsp101 in WT and transgenic *B. napus* (BL-2, BL-16, BL-19, BL-35) seedlings after exposure to 45 °C for 4 h, and to 45 °C for 4 h followed by recovery at 22 °C for 20 h were determined by western blotting. Coomassie blue-staining of ribulose-1,5-bisphosphate carboxylase/oxygenase (Rubisco) was used as a loading control.

### Fungal isolates

*L. maculans* virulent strain DAOM 194244 (Canadian Collection of Fungal Cultures, Ottawa, Canada) was cultured on 20% V8-juice agar containing 100 mg streptomycin sulfate under continuous fluorescent light^59^. *S. sclerotiorum* isolate SS01 (provided by Dr. Stephen Strelkov, University of Alberta) was cultured on solid potato dextrose agar (PDA) media (Himedia, India) at room temperature under continuous light. After 3 days, agar plugs of 2 mm diameter were removed with a sterile cork borer from the leading edge of the mycelia and grown on fresh PDA plates for an additional 2 days. Agar plugs of 5 mm diameter from the latter culture were used for inoculating plants.

### Fungal inoculation assays

Pycnidio spores from a *L. maculans* culture were collected in sterile water, the suspension was filtered through sterile cheesecloth, and the spore concentration was adjusted to 1 × 10^7^ spores mL^−1^. Each of the four cotyledon lobes of a seedling was inoculated with 10 μL of either sterile water (mock) or pycnidiospore suspension. After 4 h at room temperature, the seedling was covered with a polyethylene bag for 48 h and transferred to a growth chamber. The lesion diameter was measured at 7 dpi. The experiment was repeated three times (n = 5 seedlings/genotype).

Fully expanded 3^rd^ and 4^th^ leaves were excised, surface-sterilized with 75% ethanol and rinsed 3X with sterilized water. Leaves were placed on wet Whatman filter paper in petri dishes and the petiole bases were covered with water moistened cotton ball. *S. sclerotiorum* agar plugs were placed on the adaxial surface of the leaves and the lesion diameter was measured at 2 and 3 dpi. The experiment was repeated three times (n = 20 leaves/genotype).

*S. sclerotiorum* agar plugs were fixed with parafilm on the adaxial surface of the 3^rd^ and 4^th^ leaves of *B. napus* seedlings. Each plant was covered with a polyethylene bag for 48 h. The lesion diameter was measured at 2 and 3 dpi. The experiment was repeated three times (n = 10 leaves/genotype).

### Spider mite feeding assay

Spider mites rearing and leaf damage analysis were performed as described in Zhurov *et al.*^60^. The 2^nd^ and 3^rd^ leaves of 14-day-old seedlings were inoculated with 500 starved mites and damage was assessed after 4 days of feeding.

### Statistical analysis

All growth and stress response data, unless stated otherwise, were analyzed with the computer software SPSS (SPSS Inc., Version 16.0). Significance of differences was analyzed by one-way analysis of variance (ANOVA). Comparison among treatment means was done by the Least Significant Difference (LSD) multiple-comparison test.

### Microarray analysis

Microarray experiment details are mentioned in Supplemental methods (Methods S2). The list containing differentially expressed genes were categorized into functional groups using MapMan (version 3.1.0) http://mapman.gabipd.org/web/guest/mapman with some changes made according to information in TAIR. Genes maximally induced by BR were identified using AVT http://www.weigelworld.org/resources/microarray/AtGenExpress/.

### Identification of BES1/BZR1 sites

BES1/BZR1 binding profiles were downloaded from JASPAR and AtcisDB and scanned against 1 kb upstream sequences of Arabidopsis genes (TAIR) using the FIMO tool3 with the default p-value threshold of 1e-4.

## Additional Information

**How to cite this article**: Sahni, S. *et al.* Overexpression of the brassinosteroid biosynthetic gene *DWF4* in *Brassica napus* simultaneously increases seed yield and stress tolerance. *Sci. Rep.* **6**, 28298; doi: 10.1038/srep28298 (2016).

## Supplementary Material

Supplementary Information

## Figures and Tables

**Table 1:**
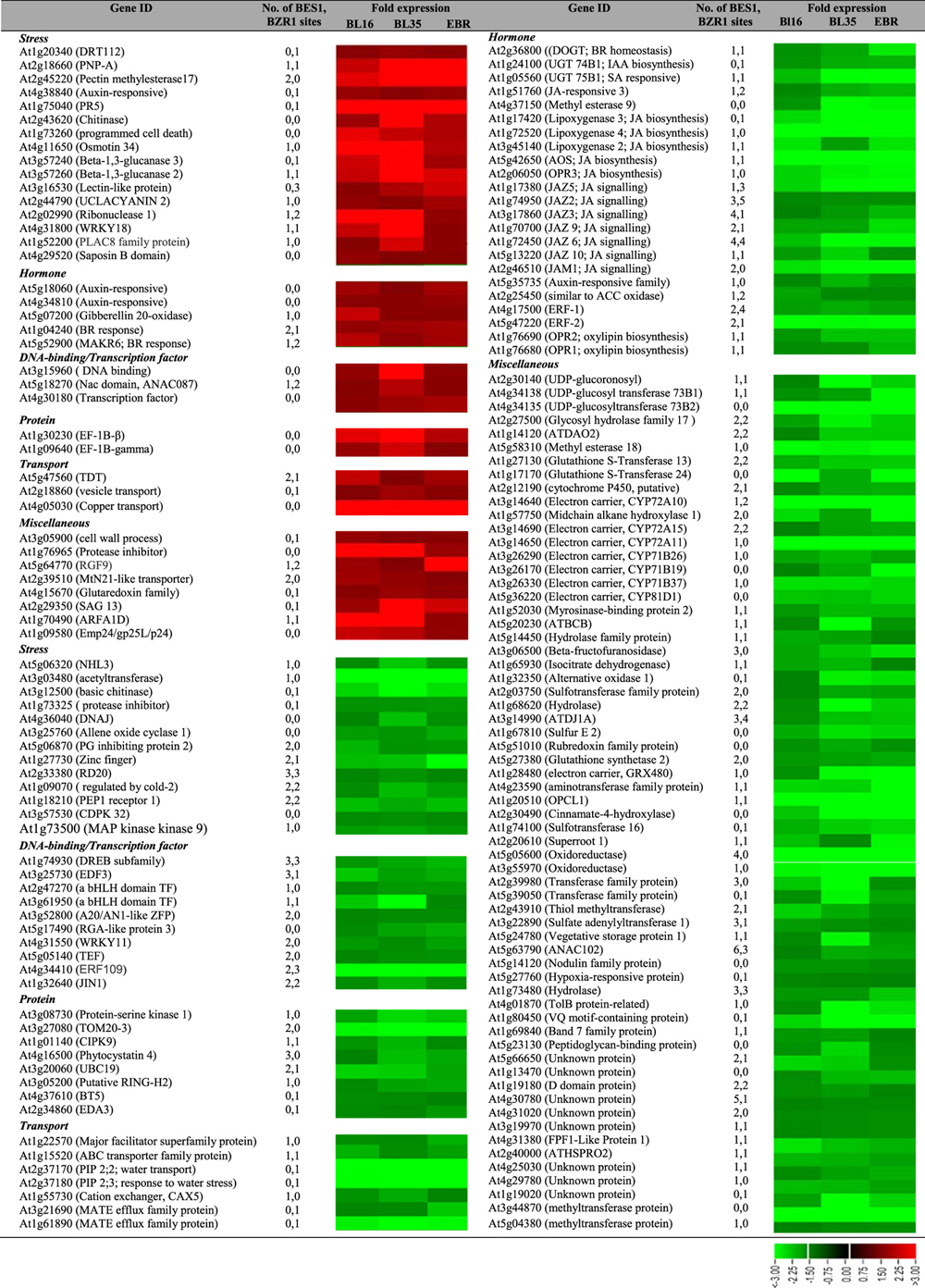
Upregulated and downregulated genes common to all three samples.

**Table 2:**
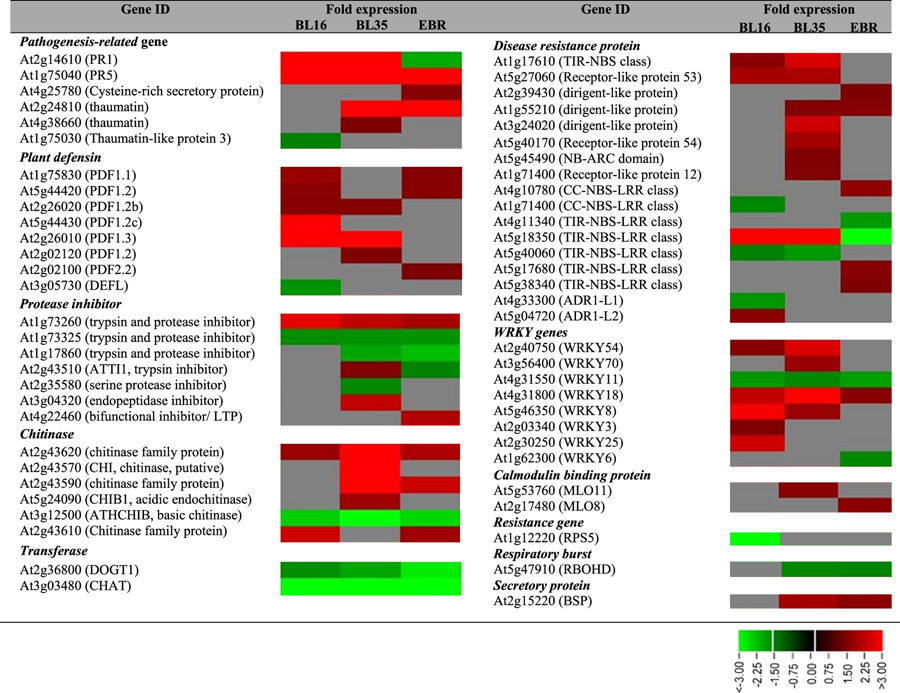
Differentially expressed defense-related genes in the three samples.
