# Supplementary material for: Overexpression of the brassinosteroid biosynthetic gene DWF4 in Brassica napus simultaneously increases seed yield and stress tolerance
Source: Sci Rep. 2016 Jun 21;6:28298. doi: 10.1038/srep28298 (PMC4915011; doi:10.1038/srep28298)
Supplement: Supplementary Information [file srep28298-s1.pdf]

Overexpression of the brassinosteroid biosynthetic gene *DWF4* in *Brassica napus* simultaneously increases seed yield and stress tolerance

Authors

Sangita Sahni[1+], Bishun D. Prasad[1+], Qing Liu[2], Vojislava Grbic[1], Andrew Sharpe[3], Surinder P. Singh[2], and Priti Krishna[1,4*]

[1]Department of Biology, The University of Western Ontario, London, ON, N6A 5B7, Canada

[2]Commonwealth Scientific and Industrial Research Organization, Agriculture, PO Box 1600, Canberra, ACT 2601, Australia

[3]National Research Council Canada, Plant Biotechnology Institute, 110 Gymnasium Place, Saskatoon, SK S7N 0W9, Canada

[4]School of Environmental and Rural Sciences, University of New England, Armidale, NSW 2350, Australia

[+]These authors contributed equally to this work.

***For correspondence (e-mail pkrishn2@une.edu.au)**

Supplementary information:

Supplementary Methods:

Methods S1

RT-PCR and qRT-PCR

qRT-PCR analysis was carried out using qScript^{TM} cDNA SuperMix (Quanta BioSciences) in a Rotor-Gene RG-3000 real-time thermal cycling system (Corbett Research). Three independent biological replicates were used with gene-specific primers (Table S4). Data were analyzed using Rotor-Gene 6.0.16 software. Values were normalized against *Actin* and fold-change was calculated using the $2^{-\Delta\Delta Ct}$method.

Methods S2

Microarray Hybridization and Scanning

WT and transgenic (BL16 and BL35) seedlings were grown for 2 weeks on MS medium in Magenta vessels. An additional set of WT seedlings were grown on MS medium supplemented with 1 μM EBR (Dhaubhadel *et al.,* 1999). Shoot tissue above the medium was harvested and total RNA was extracted using the Plant RNeasy mini kit (Qiagen). RNA amplification, labeling with Cy3- or Cy5-dCTP dyes (GE Healthcare) and probe fragmentation was carried out using Ambion Amno Allyl Message Amp II RNA amplification kit (Ambion). A dye swap (Cy3/Cy5) experiment was performed for each biological replicate. A spotted 15,000, 50-mer *B. napus* oligo array, previously developed at the Agriculture and Agri-Food Canada (Stasolla *et al.,* 2008), was hybridized with the Cy5- and Cy3-labelled probe pairs. Labeling, hybridization and post-hybridization washing were conducted according to the protocol for Corning epoxide slides ("long-oligo" protocol with 25% formamide; Corning Inc.). Hybridization was carried out at 37°C for 17 h in a MAUI hybridization station (BioMicro Systems). Post-hybridization washes were carried out once in 2x SSC, 0.1% SDS at 37°C for 5 min, twice in 1x SSC at room temperature for 2 min, and twice in 0.1x SSC at room temperature for 1 min. Slides were scanned with the Vers Array Chip Reader laser scanner (BioRad). Image analysis and feature extractions were performed with Array Pro Analyzer software (Media Cybernetics Inc.). Initial data processing was performed using tools available in BASE database (http://base.thep.lu.se). Background intensities were removed by subtracting median background from median foreground spot intensities. Signal intensity data were normalized using a pin-based Lowess normalization.

Intensity data for each reporter pair in the dye-swap experiments was merged by calculating the geometric mean of ratios per reporter pair.

Methods S3

Lipid Extraction and FAMEs Preparation and Analysis

Lipid extraction and fatty acid analysis in *B. napus* seeds were carried out according to Siloto *et al.* (2006). For Arabidopsis seeds, 5 mg of dry seeds were incubated with 0.7 mL 2% H_2SO_4/MeOH, 18 µL 0.2% BHT/MeOH and 200 µL C17:0-TAG/toluene (1 µg/µL) at 95°C for 90 min. Following extraction with 0.3 mL 0.9% NaCl (w/v), 0.5 mL hexane and centrifugation at 1500x g for 5 min, the upper phase containing total fatty acid methyl esters (FAMEs) was analyzed by an Agilent Technologies 6890N GC equipped with a 60-m BPX70 column using helium as carrier gas. The column temperature was programmed as an initial 170°C for 1 min, ramping to 200°C at 5°C/min, 210°C at 2.5°C/min, and finally to 240°C at 10°C/min with holding for 3 min. Peaks were identified based on comparison of retention time with standard FAME and quantified with Agilent Technologies Chem Station software (Rev B.03-02-SR2). The mass of individual FAME was calculated on the basis of internal standard mass and converted into moles using molecular weights of FAMEs. Total masses of glycerol moieties and fatty acyl moieties were summed to calculate the total amount of TAG and percentage of oil in seed.

Methods S4

Hormone Analysis

Leaf tissue of WT, VC and transgenic seedlings grown for 2 weeks on MS medium, and WT grown on MS medium plus 1 µM EBR, was collected, frozen in liquid nitrogen, and stored at -80°C until used. Frozen leaf material (~500 mg) was analyzed for SA and JA levels at the hormone profiling facility at the Plant Biotechnology Institute, National Research Council, Saskatoon http://www.pbi.nrc.ca/ENGLISH/technologyplatforms/plant-hormone-profiling.htm. The analysis was performed by high performance liquid chromatography electrospray tandem mass spectrometry (HPLC-ES-MS/MS) using deuterated internal standards (1 ng/ L of 3,4,5,6-*d*4-2-hydroxybenzoic acid and 0.5 ng/ L of 2,2-*d*2-jasmonic acid) and external standards (100 ng of 1,2,3,4,5,6-13*C*6-2-hydroxybenzoic acid and 50 ng of 12,12,12-*d*3-jasmonic acid).

Supplementary Figures:

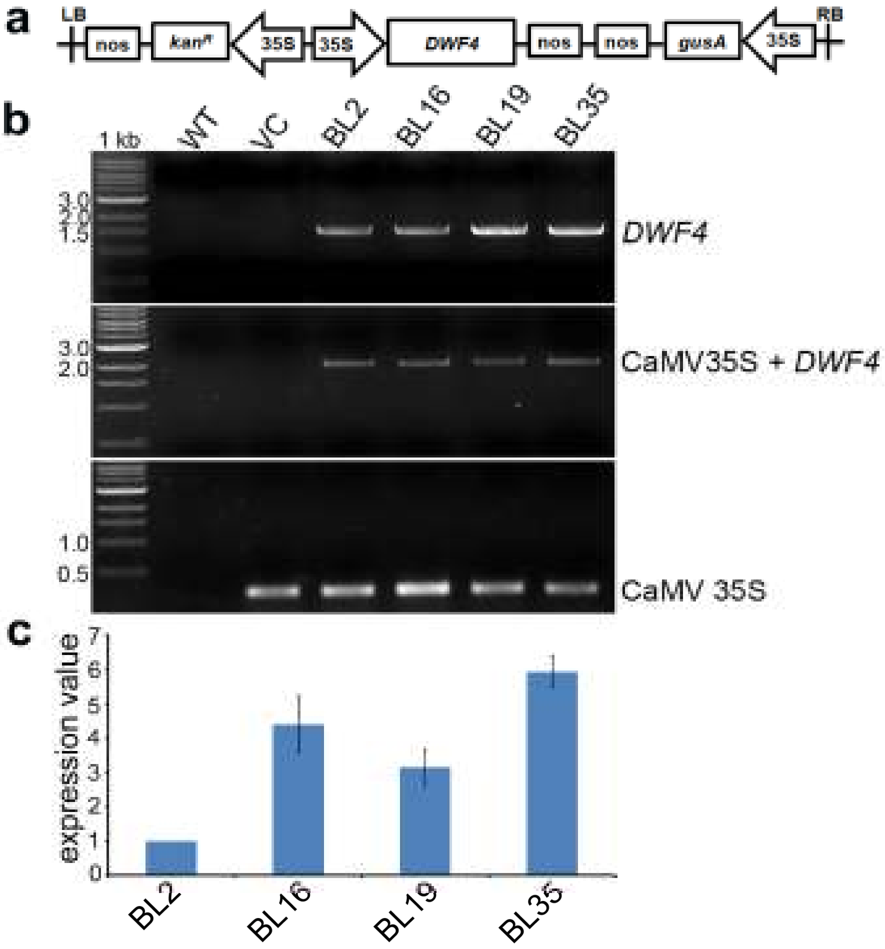

Figure S1. Schematic of the transgene construct and its verification in transgenic lines.

(a) Schematic diagram of the transgene construct (pCambia2301-*DWF4*) used for plant transformation. LB, left border; RB, right border; 35S, Cauliflower mosaic virus 35S promoter; nos, nopaline synthase terminator; *Kan*^{R}, kanamycin resistance gene; *gusA*, β-glucuronidase gene.

(b) PCR amplification of the transgene from genomic DNA of WT, VC and transgenic lines using different primer pairs (AtDWF4-F + AtDWF4-R, CaMV35S-F + AtDWF4-R, and CaMV35S-F + CaMV35S-R).

(c) qRT-PCR analysis of *AtDWF4* expression in different transgenic lines. Expression levels are presented relative to transgenic line BL2.

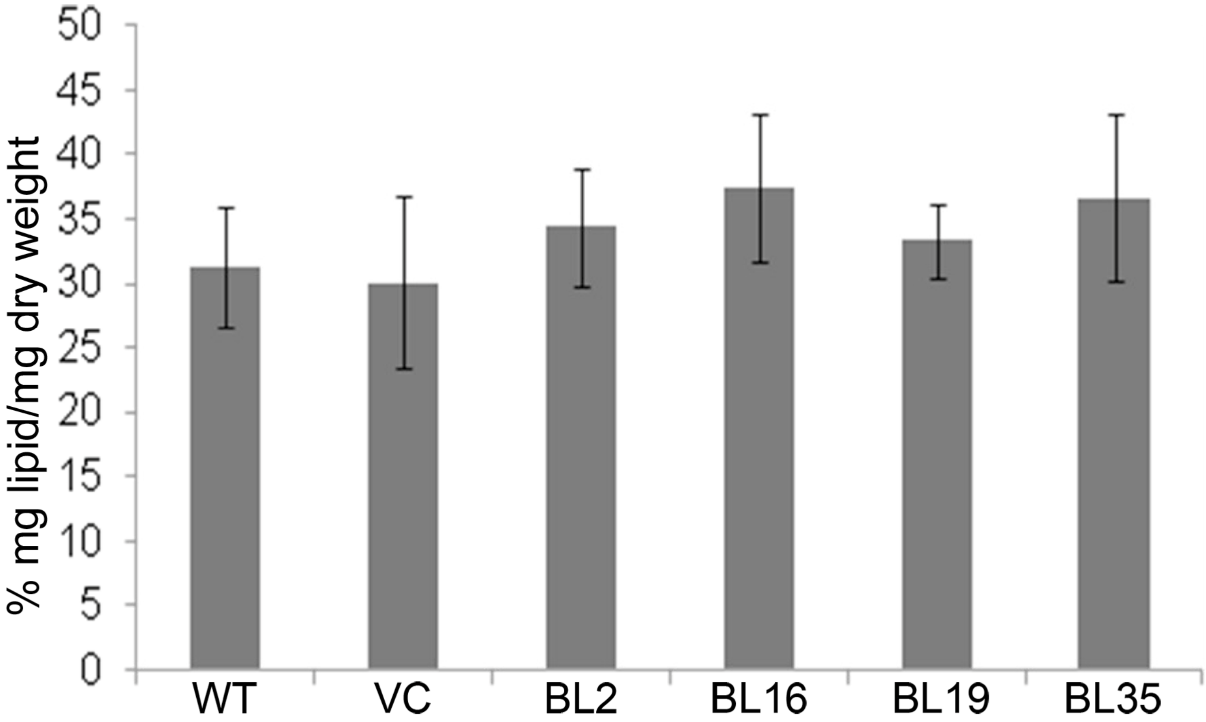

Figure S2. Lipid content in *B. napus* seeds. Percent lipid content (% mg lipid/mg dry weight) in mature *B. napus* seeds of WT, VC and transgenic lines. Results are representative of 3 independent samples of each genotype. Error bars represent standard deviation (SD) of mean for three replicates.

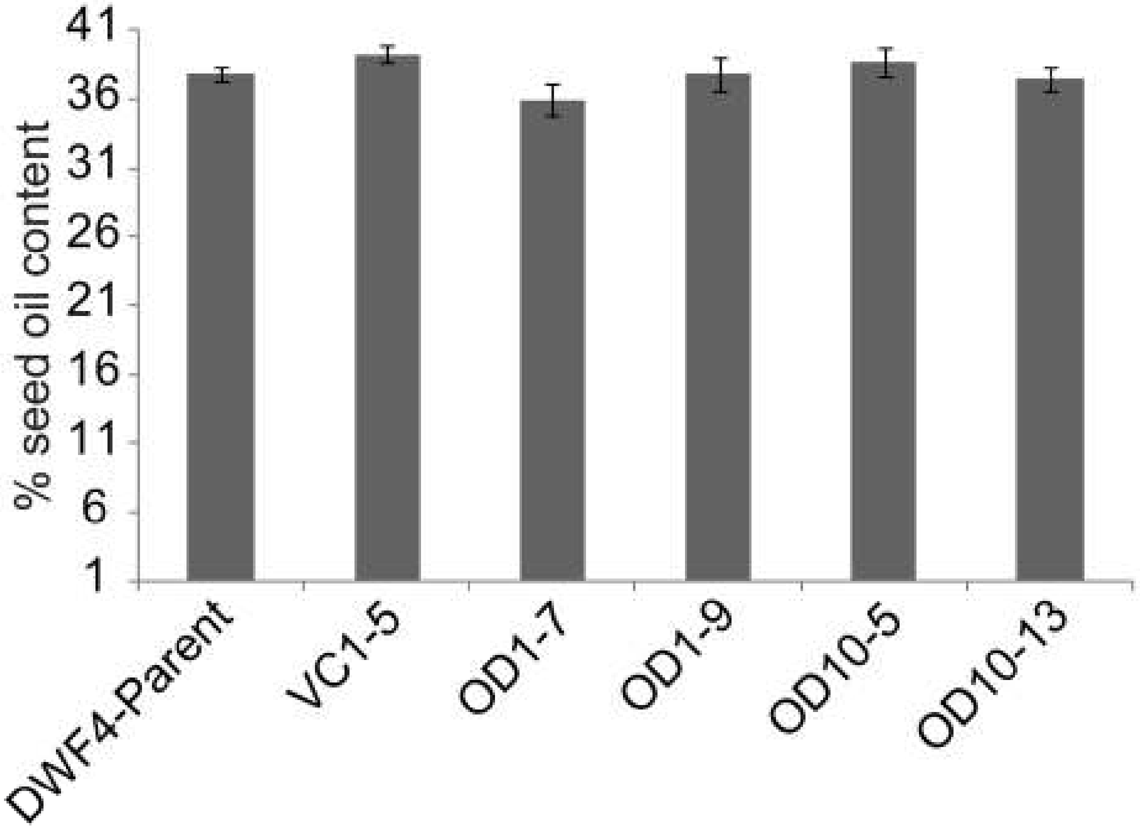

Figure S3. Lipid content in Arabidopsis seeds; WT, vector control (VC1-5) and transgenic lines OD1-7, OD1-9, OD10-5 and OD10-13. The data were from eight replicates and presented as means ± SE.

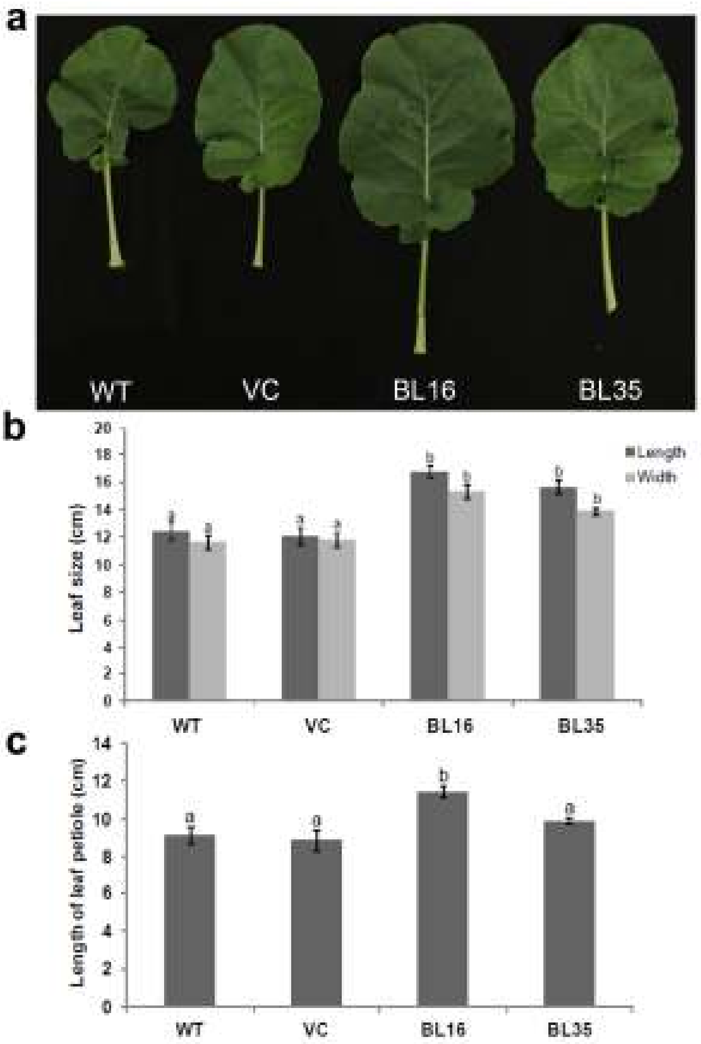

Figure S4. Phenotypes of leaves of plants grown in hydroponic culture.

(a) Phenotypes of the leaves of WT, VC and transgenic lines grown in hydroponic culture for 30 d.

(b) Measurements of the length and width of the 4th leaf.

(c) Measurements of the length of the 4th leaf petiole. Results are representative of two biological experiments.

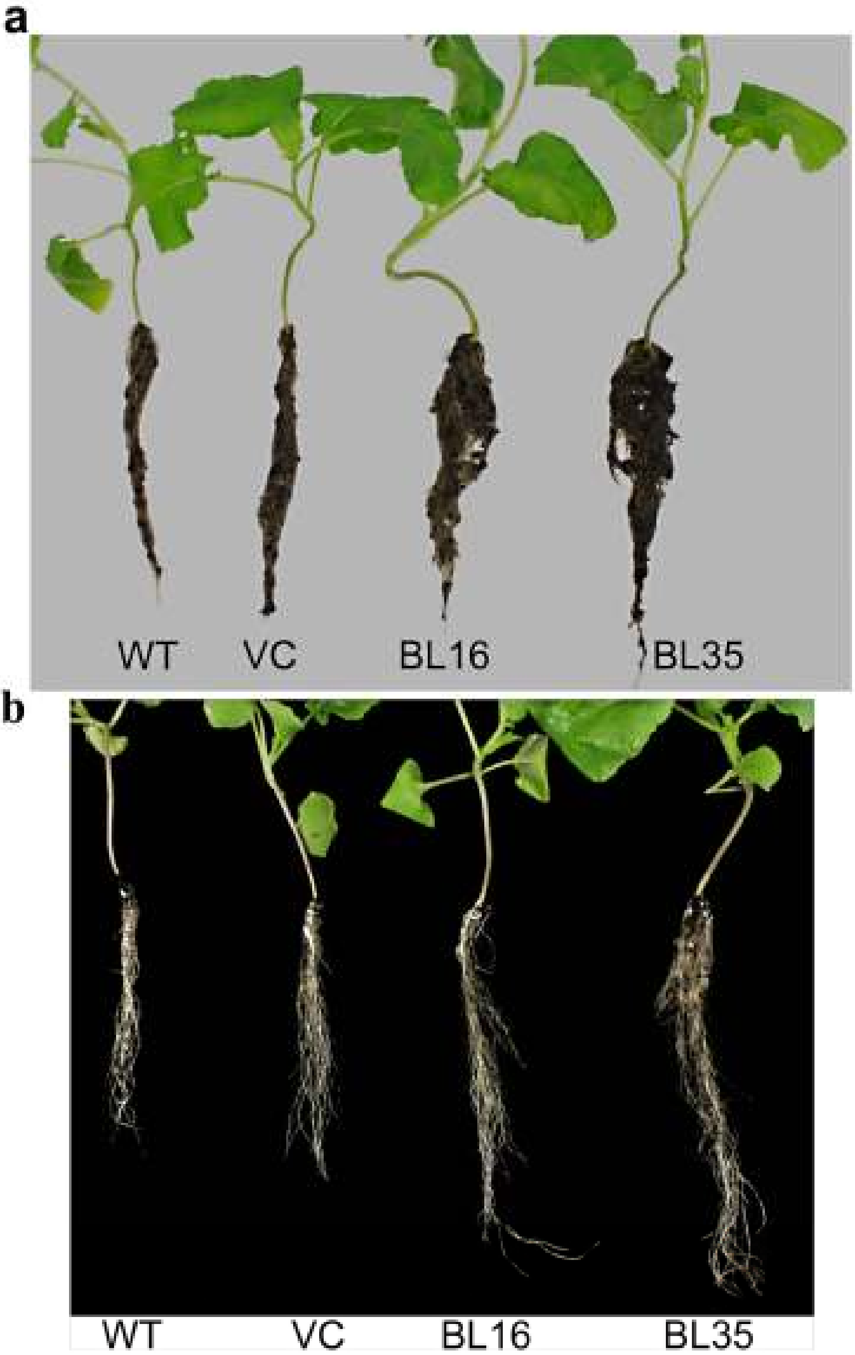

Figure S5. Phenotypes of the roots of WT, VC and transgenic lines grown for 30 d in pots with Promix BX soil. (a) Photograph of roots with attached soil. (b) Photograph of roots after removing most of the soil.

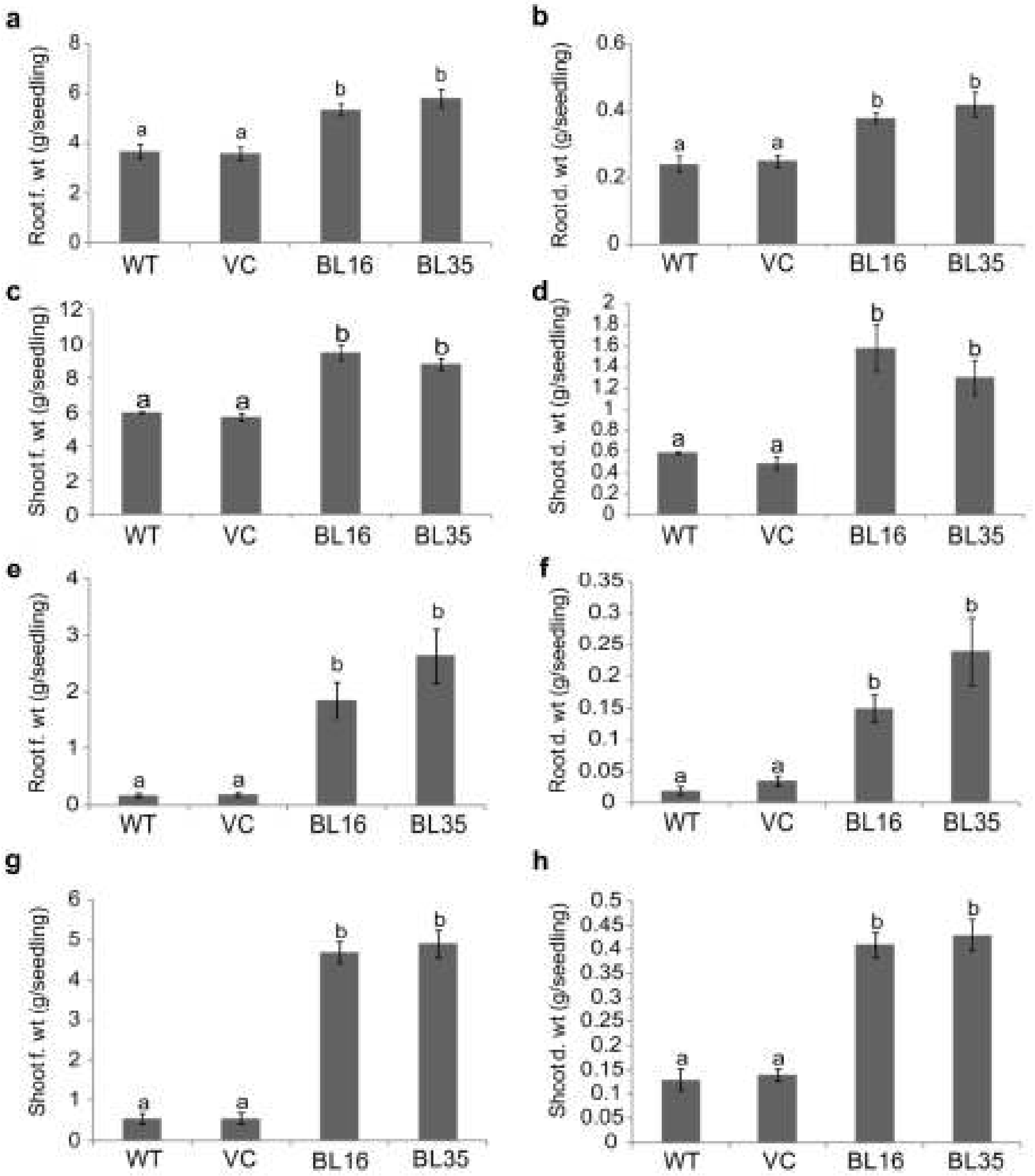

Figure S6. Effect of dehydration stress on fresh and dry weights of shoots and roots of WT, VC and transgenic lines. (a-d) Root and shoot weights of plants grown for 47 days under normal conditions. (e-h) Root and shoot weights of plants subjected to dehydration stress for 12 d followed by re-watering for 7 d. Results are representative of three biological experiments.

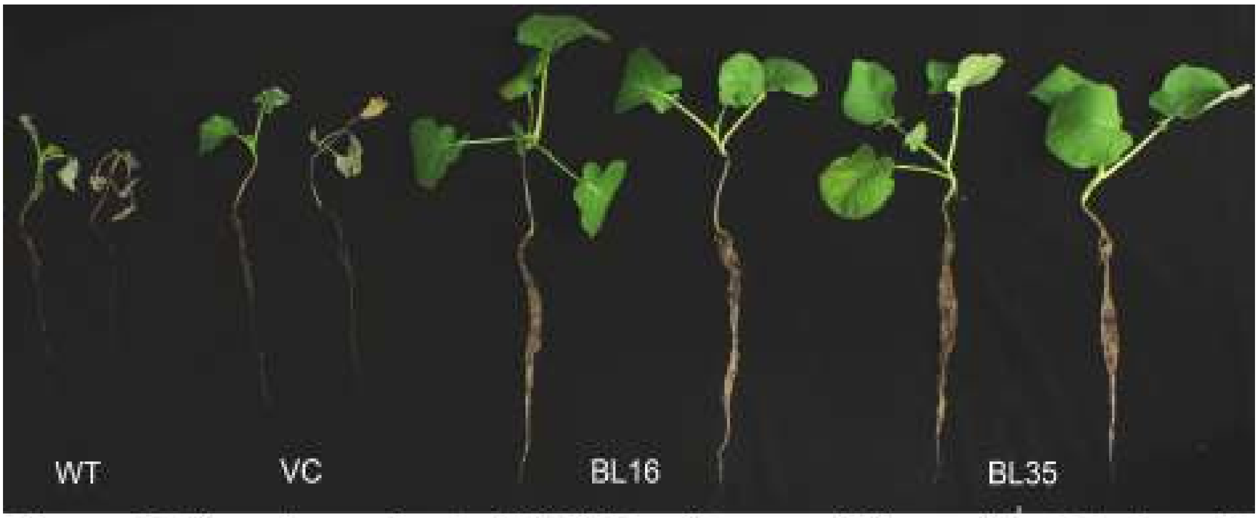

Figure S7. Phenotypes of roots of WT, VC and transgenic lines subjected to drought stress for 12 d followed by re-watering for 7 d.

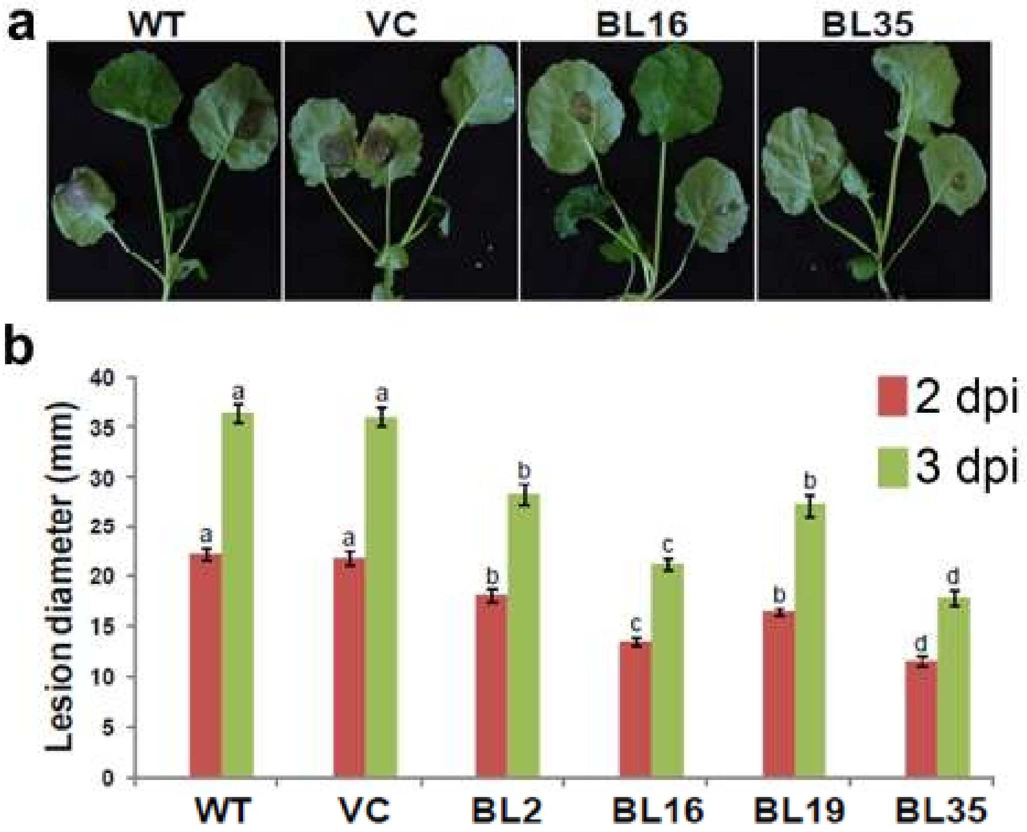

Figure S8. Test for resistance to *S. sclerotiorum* in whole seedling assay. Necrotic lesions (a) and lesion diameter (b) on leaves of whole seedlings challenged with *S. sclerotiorum*. Results are representative of three independent experiments.

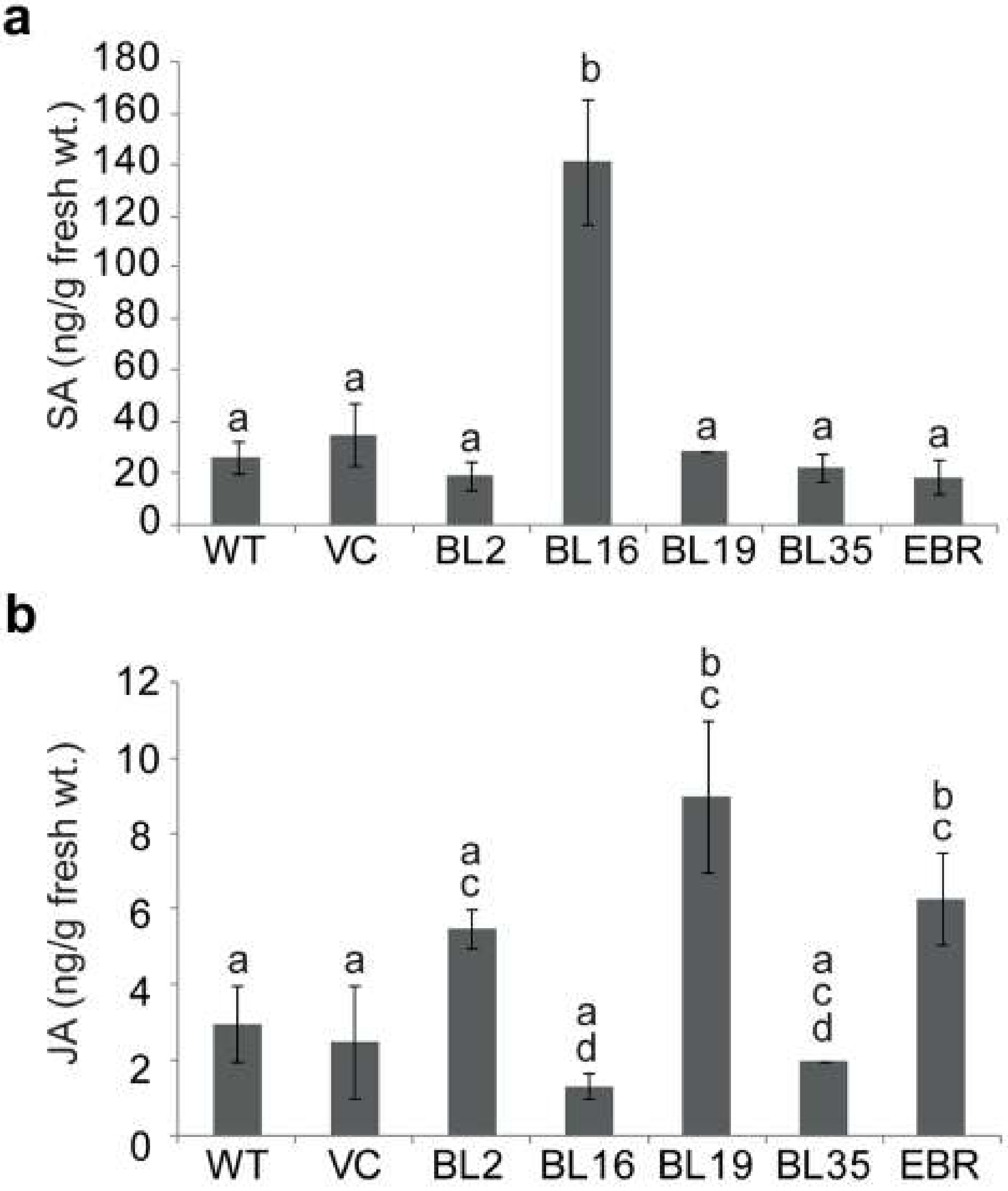

Figure S9. SA (a) and JA (b) contents in leaves of 2-wk-old WT, VC, transgenic lines and EBR-treated *B. napus* seedlings grown on MS medium. Error bars represent standard error (SE) of mean for three (WT, VC, BL16 and BL35) and two (BL2 and BL19) replicates.

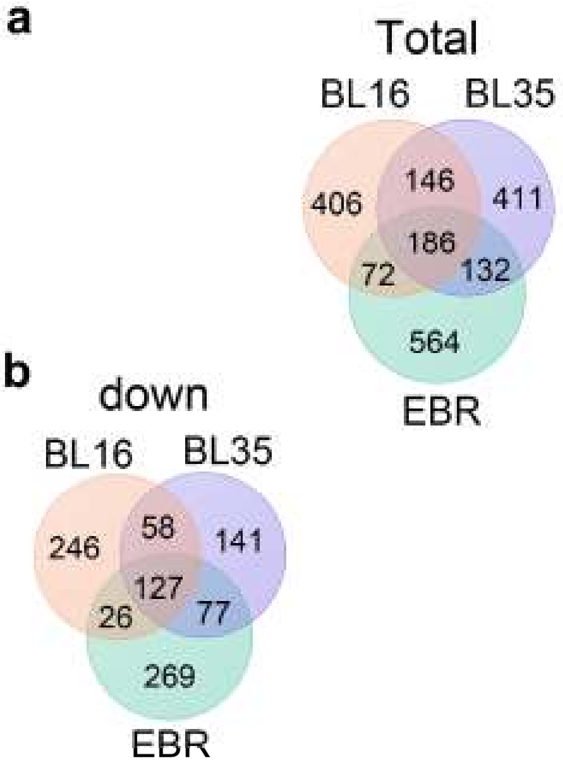

Figure S10. Venn diagram summary of differentially expressed (≥1.5-fold) genes. Venn diagram representing overlap between total numbers of differentially expressed genes (a) and overlap between down and up-regulated genes (b) in transgenic BL16, BL35, and EBR-treated seedlings.

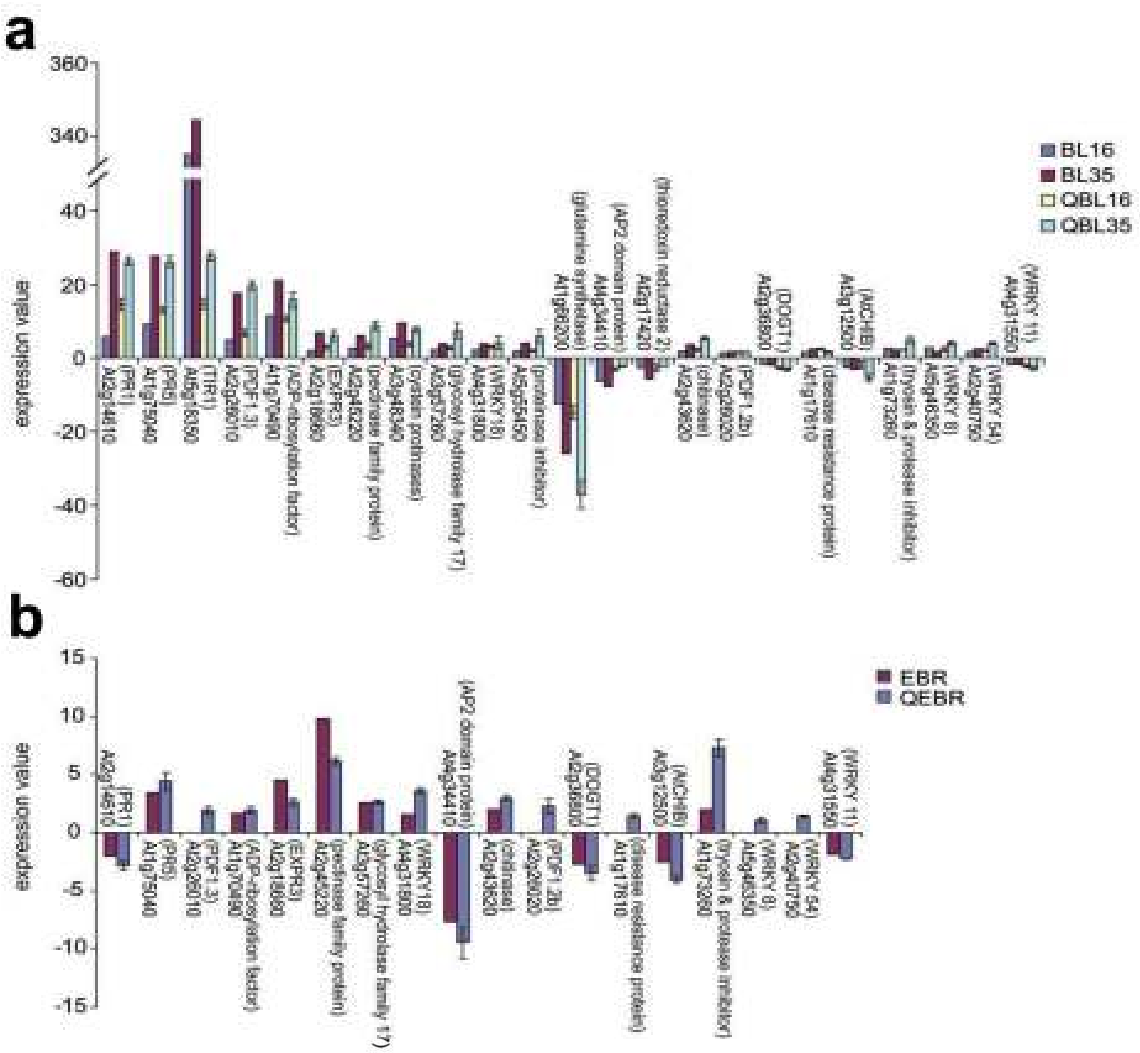

Figure S11. Validation of microarray data by qRT-PCR.
(a) Gene expression changes in BL16 and BL35 relative to WT.
(b) Gene expression changes in EBR-treated compared to untreated seedlings. QBL16, QBL35 and QEBR represent qRT-PCR data, while regular names represent microarray data. Error bars represent standard error (SE) of mean for three replicates.

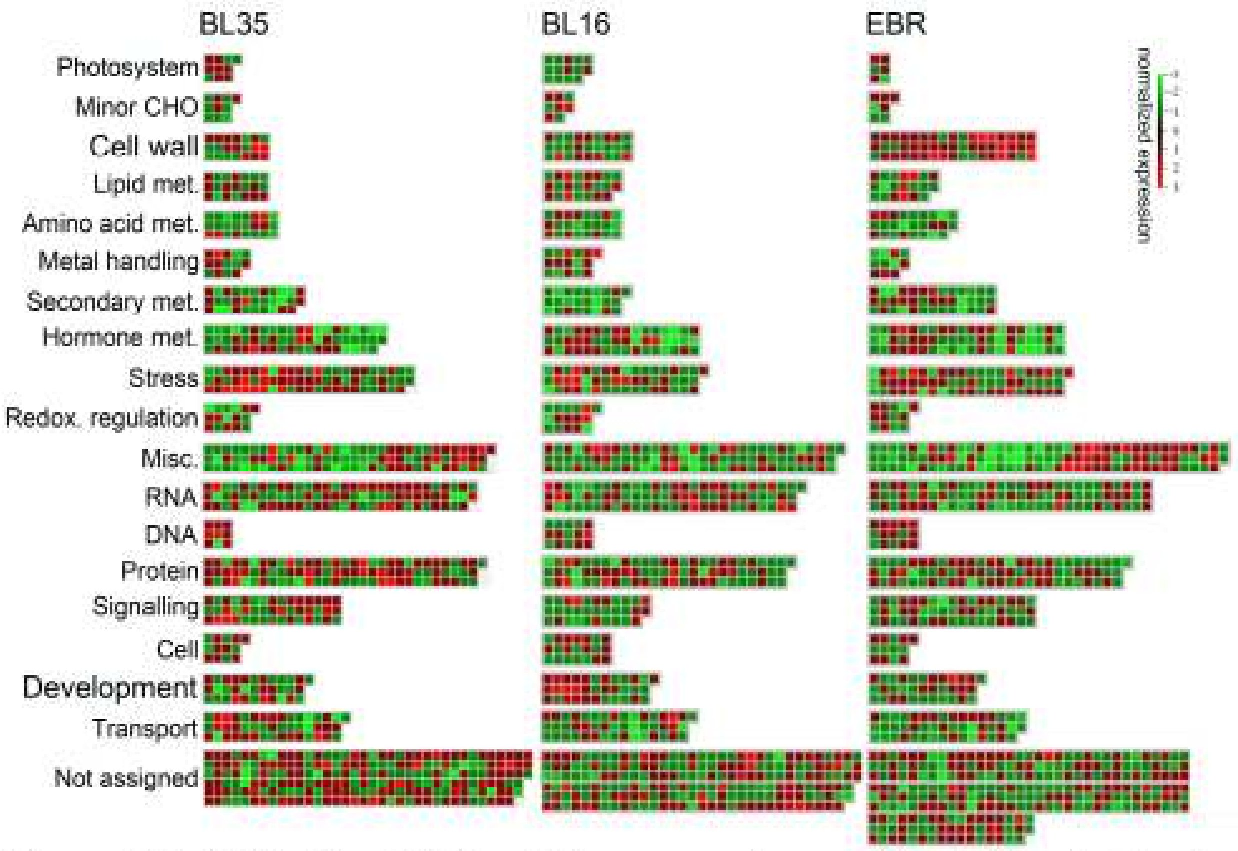

Figure S12. Distribution of differentially expressed genes into functional categories (BINS) with MapMan. Each square represents a gene and the color of the square is indicative of the normalized expression level in transgenic lines BL35 and BL16, and EBR-treated seedlings compared to WT.

Supplementary Tables:

Table S1. Fatty acid composition of mature *B. napus* seeds. The % relative fatty acid species derived from the total lipid of the WT and VC in comparison to the transgenic lines. The major fatty acid species, denoted by the shaded boxes, constitute greater than 95% of the fatty acid species. For statistical analysis a two tailed t-test assuming equal variance with a p=0.01 was performed.

**Fatty acid**	**Sample % relative fatty acid (±SD)**					
	**WT**	**VC**	**BL2**	**BL16**	**BL19**	**BL35**
**C14:0**	0.20±0.11	0.22±0.13	0.22±0.05	0.16±0.01	0.18±0.06	0.27±0.19
**C16:0**	5.69±1.05	5.88±0.59	5.60±0.49	5.09±0.29	4.32±1.86	5.21±0.42
**C16:1**	0.22±0.11	0.32±0.04[b]	0.26±0.06	0.24±0.03[b]	0.24±0.01[b]	0.23±0.02[b]
**C18:0**	2.47±0.57	3.24±0.24[b]	2.57±0.40[b]	2.60±0.35[b]	2.51±0.20[b]	2.64±0.51
**C18:1**	63.75±3.23	59.95±3.21[b]	65.52±4.41	64.80±1.03[b]	65.07±2.07[b]	64.80±2.31
**C18:2**	18.39±1.25	21.21±2.27	17.50±2.80	18.45±1.41	18.89±1.63	18.33±2.33
**C18:3**	6.91±2.56	6.15±0.60[b]	4.94±1.77	5.52±0.73	4.90±0.93	4.94±0.69[b]
**C20:0**	0.63±0.14[a]	0.88±0.07[a]	0.85±0.12	0.79±0.14	0.81±0.08	0.83±0.12
**C20:1**	1.14±0.08	1.07±0.07[b]	1.09±0.07	1.12±0.12	1.13±0.04	1.19±0.04[a]
**C20:2**	ND* [a]	ND* [b]	0.11±0.16	0.15±0.13	0.11±0.03[ab]	0.22±0.07[ab]
**C22:0**	0.30±0.16[a]	0.52±0.06	0.54±0.07[a]	0.45±0.12	0.44±0.06	0.49±0.09
**C24:0**	ND* [a]	0.17±0.18	0.40±0.07[a]	0.27±0.15[a]	0.28±0.05[a]	0.35±0.05[a]
**C24:1**	0.31±0.15	0.39±0.05[b]	0.41±0.10	0.36±0.14	0.32±0.03[b]	0.50±0.16

[*] ND= not detected

[a] significant difference between WT and VC or BL lines (p=0.01 (2), 6=3.169)

[b] significant difference between VC and BL lines (p=0.01 (2), 6=3.169)

Table S2. Fatty acid composition of mature Arabidopsis seeds expressing *AtDWF4* under the control of a seed-specific oleosin promoter. The transgenic lines were obtained from independent transformation events. All data are averages of eight independent measurements ± SE.

**Fatty acid**	**Fatty Acid Composition (% of total)**					
	**DWF4-P**	**VC1-5**	**OD1-7**	**OD1-9**	**OD10-5**	**OD10-13**
**C16:0**	7.7±0.1	7.8±0.1	7.8±0.3	7.8±0.3	7.8±0.1	7.8±0.1
**C16:1**	0.3±0.0	0.3±0.0	0.3±0.0	0.3±0.0	0.3±0.0	0.3±0.0
**C18:0**	3.3±0.1	3.3±0.1	3.3±0.1	3.2±0.2	3.2±0.1	3.3±0.1
**C18:1**	15.7±0.4	15.5±0.6	16.2±1.7	16.6±1.5	15.7±0.9	16.5±0.7
**C18:1Δ11**	1.7±0.1	1.7±0.1	1.7±0.1	1.8±0.2	1.8±0.0	1.8±0.1
**C18:2**	28.6±0.4	28.6±0.4	28.9±0.5	29.0±0.8	29.1±0.2	29.1±0.2
**C18:3n3**	15.5±0.5	15.7±0.4	14.8±0.7	14.8±0.9	15.3±0.4	14.6±0.5
**C20:0**	2.4±0.1	2.3±0.1	2.4±0.1	2.1±0.2	2.2±0.1	2.4±0.1
**C20:1**	18.8±0.4	18.9±0.3	18.8±0.4	18.7±0.7	18.7±0.2	18.4±0.2
**20:1iso**	1.8±0.1	1.8±0.1	1.8±0.1	1.8±0.1	1.8±0.1	1.8±0.1
**20:2n6**	1.7±0.0	1.7±0.1	1.6±0.1	1.6±0.1	1.7±0.1	1.6±0.1
**20:3n3**	0.4±0.0	0.4±0.0	0.3±0.0	0.3±0.0	0.4±0.0	0.3±0.0
**C22:0**	0.3±0.0	0.3±0.0	0.3±0.0	0.3±0.0	0.3±0.0	0.3±0.0
**C22:1**	1.7±0.1	1.7±0.0	1.7±0.1	1.6±0.1	1.7±0.0	1.6±0.0

Table S3. Genes maximally upregulated by BR in AtGenExpress dataset.

S. No	Gene ID, Protein type and function	BL16	BL35	EBR
1.	At3g57240, Beta-1-3-glucanase 3, defense response			
2.	At1g51805, Leucine-rich repeat protein kinase family protein, protein phosphorylation			
3.	At3g06180, Ribosomal protein L34e superfamily protein, translation			
4.	At3g28510, P-loop containing NTP hydrolases superfamily protein, systemic acquired resistance			
5.	At3g26490, Phototropic-responsive NPH3 family protein, response to light stimulus			
6.	At3g09440, Heat shock protein 70 (Hsp70) family protein, response to heat			
7.	At1g35710, Protein kinase family protein with leucine-rich repeat domain, defense response			
8.	At1g10550, Xyloglucan:xyloglucosyl transferase 33 (XET), cell wall modification			
9.	At2g21780, Unknown protein, fatty acid catabolic process			
10.	At2g30250, ATWRKY25, transcription factor, response to numerous abiotic stresses			
11.	At5g26920, Calmodulin-binding protein, defense response			
12.	At4g04540, Cysteine-rich receptor-like protein kinase 39 (CRK39), protein phosphorylation			
13.	At4g02330, Pectin methylesterase 41 (ATPME41), response to brassinosteroid stimulus, cold, fungus			

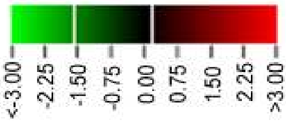

Table S4. List of primers. Sequences of primers used in quantitative RT-PCR (qPCR) analysis.

S.No	Name of Primer	Primer sequence (5'—3') Forward (F)	Reverse (R)
1.	At2g14610	GACCGACTAAGAGGCGACTG	TAATTGCCCCGAGGATCATA
2.	At1g75040	AGATCGGATAGCTAGGGTT	GGCCGCTCTAGAACTAGTG
3.	At5g18350	CTATAGATGCAAATGGCTGTT	TGCAAGCTTTGAATCTCAAGG
4.	At2g26010	CGTCCATCATCACCCTTCTC	TTGGTAGATGAATCGCATGG
5.	At1g70490	AGCGTCACTGGTACATTCAGAG	CTTCATTACGCCTTGCCAGC
6.	At2g18660	ATGCCGGCAACGTCCGCG	TGATTTCACCAAGGACCATCC
7.	At2g45220	GGATGGCTTGAGTGGAATGG	ATTTGACCCGACCTGAAGTGG
8.	At3g48340	TACAGTTGCAGCCGTTGAAG	CAAATGCAATGTCCATGAGG
9.	At3g57260	ATGAGGATCGGTTTGAGAGG	CACTGCTTTACTAGCCCTTGC
10.	At4g31800	GCGGCTGCTATATCTGGGAG	GGCCACTCCTATTTCCAGGG
11.	At5g55450	ACACCGACACAAACGACTTG	GAGACATTCAAGATCAGCGG
12.	At1g66200	GTTTTGGGGTTTGTGATTGG	TCAAGGCCTTTTCAGATCG
13.	At4g34410	GAGTGCGAATGCAAGTACGAG	TGCCTGAATCCGAAGAATCTC
14.	At2g17420	CTTCAGGAACAAGCCTCTGG	TTCGACGCCCTAAACGTATCC
15.	At2g43620	TTTGGGATCACTCCTGGAAC	TTAACTCACACGCGCAAGAC
16.	At2g26020	AATGGTGAAAGCGCAGAAG	GTTGCAAGATCCATGTCGTG
17.	At2g36800	GGGAAAATAGGTGTGTTGGTG	TCCAAGCTCTTTGGCTCTTC
18.	At1g17610	TGCAGGTTTGGAAGATTATGG	ACATGGGACAAGTGTCTCCTC
19.	At3g12500	AACGATAACGCTTGTCCTGC	ACCAGTGGTTTCGTGGGAAG
20.	At1g73260	CCTAGAGTTGCGTTCGTTCC	CGAAATACTGCTGCCTCTCC
21.	At5g46350	TCTTCACGTTGCACTTCTGC	TCGAAGACGGCTATCGTTGG
22.	At2g40750	AGGTATGGTGAAGCAGGAGG	TCTTGCCAAACCAATGACAAG
23.	At4g31550	TGTGGAACGAGCTTTGGATG	ATCATGCCGAAGCAAACACC
24.	BnRD20	CAACATTCACAAAGCCAAGCA	TCTCGAGATTAACCGGGACGTA
25.	BnRD22	CGGGTGCTGCGATTTTGTAC	GAAAAGAGTTGGGGGAGAGG
26.	UBQ10	TGACAACGTGAAGGCCAAGATCC	ATACCTCCACGCAGACGCAACAC
27.	AtDWF4	ATGTTCGAAACAGAGCATC	TTACAGAATACGAGAAACC
28.	CaMV	CTCGGATTCCATTGCCCAGCTAT	TTGCGAAGGATAGTGGGATTGTGC
29.	*ACTIN*	ATCACCATCGGAGCTGAG	GAAGCATTTCCTGTGGACG

References

Dhaubhadel, S. *et al.* Treatment with 24-epibrassinolide, a brassinosteroid, increases the basic thermotolerance of Brassica napus and tomato seedlings. *Plant Mol Biol.* **40**, 333-342 (1999).

Siloto, R.M. *et al*. The accumulation of oleosins determines the size of seed oilbodies in Arabidopsis. *Plant Cell* **18**,1961-1974 (2006).

Stasolla, C. *et al*. Buthioninesulfoximine (BSO)-mediated improvement in cultured embryo quality in vitro entails changes in ascorbate metabolism, meristem development and embryo maturation. *Planta* **228**:255-272 (2008).
